# Integrated metabolomic and transcriptomic analyses reveal temporal dynamics of secondary metabolite accumulation in *Cibotium barometz* rhizome

**DOI:** 10.3389/fpls.2025.1702726

**Published:** 2025-12-05

**Authors:** Feng-Pan Wang, Li-Jun Chen, Shao-Rong Zhou, Meng Tang, Xuejiao Zhang, Yue-Hong Yan

**Affiliations:** 1 Key Laboratory of National Forestry and Grassland Administration for Orchid Conservation and Utilization, The Orchid Conservation and Research Center of Shenzhen, Shenzhen, China; 2 Shenzhen Dapeng Peninsula National Geopark, Shenzhen, China; 3 Shanghai Chenshan Botanical Garden (Chenshan Scientific Research Center of Chinese Academy of Sciences (CAS) Center for Excellence in Molecular Plant Sciences), Shanghai Key Laboratory of Plant Functional Genomics and Resources, Shanghai, China

**Keywords:** *Cibotium barometz*, *Gou-ji*, multi-omics integration, secondary metabolite, growth years, flavonoids

## Abstract

*Cibotium barometz* (*Gou-ji*) is a valued traditional Chinese medicinal herb with demonstrated efficacy in treating musculoskeletal and age-related conditions. The pharmacological properties of *Gou-ji* are closely associated with its phytochemical composition. However, a systematic understanding of the metabolic profile and its temporal dynamics in the rhizome—the primary medicinal part—remains limited. This study aimed to comprehensively characterize the secondary metabolite composition of *C. barometz* rhizome and elucidate its developmental regulation. Our specific objectives were to: (1) compare the metabolomic profiles of rhizomes at juvenile (YDS), intermediate (MDS), and mature (MS) stages; (2) investigate the transcriptomic basis underlying metabolite accumulation; and (3) quantify flavonoids across developmental stages. Using broadly targeted metabolomics, we identified a total of 761 secondary metabolites from *Gou-ji*, among which phenolic acids and flavonoids accounted for over 50%. Metabolite profiling revealed stage-specific shifts in accumulation patterns: phenolic acids were most enriched in YDS rhizome, alkaloids in MDS rhizome, and flavonoids in MS rhizome. In comparison with YDS or MDS rhizome, MS rhizome exhibited significant enrichment of numerous flavonoids, indicating a consistent and pronounced accumulation of flavonoids in more mature rhizome. Transcriptomic profiling further revealed upregulation of key genes involved in flavonoid biosynthesis pathways in MS rhizome, providing molecular insights into the observed metabolic changes. Furthermore, quantitative analysis confirmed higher flavonoid content in MS rhizome compared to YDS, particularly for the high-abundance flavonoids: (-)-epicatechin, apigenin-7-glucoside, naringenin-7-glucoside, vitexin, apigenin, (-)-catechin, eriodictyol, and naringenin chalcone. It supported the potential role of prolonged growth in enhancing flavonoid accumulation. These findings not only enhance our understanding of the phytochemical basis of *Gou-ji*’s medicinal efficacy but also offer valuable insights for quality assessment and optimized harvesting of this traditional herb.

## Introduction

*Cibotium barometz* (L.) J.Sm, commonly known as golden chicken fern or woolly fern, is a perennial herbaceous fern belonging to the Cibotiaceae family. The *Cibotium* exhibits a broad geographical distribution across the tropical and subtropical regions of East and Southeast Asia, the Hawaiian Islands, and Mesoamerica (Hu et al., 2023; Jiang et al., 2023). *C. barometz* is characterized by its thick, woody rhizome, which accumulates pharmacologically active secondary metabolites (Chandra, 1970; Jiang et al., 2023; Kim et al., 2023; Wu and Yang, 2009). The dry or processed rhizome of *C. barometz* have served as traditional Chinese folk medicine *Gou-ji* (Rhizoma Cibotii) for a long history since the Eastern Han Dynasty (25~220 CE). *Gou-ji* is listed in the Chinese Pharmacopoeia (2020 Edition) and other folk medicinal formulas. Its clinical applications include kidney tonification (Liu et al., 2020), strengthening the lower back (Liu et al., 2020), hepatoprotection (Li et al., 2019; Xie et al., 2017), and enhancing bone health (Chen et al., 2022; Huang et al., 2018; Huang et al., 2019; Zhao et al., 2011). These well-validated properties drive significant commercial demand of *Gou-ji* materials.

The pharmacological properties of Chinese medicinal plants primarily derive from secondary metabolites such as flavonoids, terpenoids, and phenolic acids (Nett et al., 2023; Seyedi et al., 2023; Yin et al., 2025). The aqueous or organic solvent extracts of *Gou-ji* contain phenolic acids (Kim et al., 2023), polysaccharides (Huang et al., 2018; Huang et al., 2020; Li et al., 2019), hemiterpene glucosides (Xie et al., 2017) and other bioactive compounds, which are associated with its pharmacological activity. The identification and experimental validation of bioactive components from *Gou-ji* remain a central focus of pharmacological research on this traditional Chinese herb. Despite the recognized medicinal value of *C. barometz* and the application of modern research techniques, a significant and specific knowledge gap persists. A recent 2025 review of the species underscores that a ‘systematic understanding’ of its metabolic profile is still lacking (Xu et al., 2025). This gap is particularly evident when considering the plant’s development. Chen et al. (2025) employed an integrated transcriptomic and metabolomic approach to illustrate the regulatory networks and dynamic metabolite changes in *C. barometz* leaf development. However, this valuable research contrasts sharply with the limited knowledge of the rhizome—the primary medicinal part—across its developmental stages. The critical question of how an extended growth duration influences the secondary metabolite profile in the rhizome remains entirely unexplored. This lack of a temporal perspective on the phytochemical composition of the rhizome severely limits the scientific basis for determining optimal harvest time to ensure maximum medicinal quality and efficacy.

For perennial medicinal and aromatic plants, harvesting time is considered a pivotal factor which significantly influences the quantity and quality of natural chemistry compounds (Cui et al., 2025; Hazrati et al., 2024; Li et al., 2020; Yuan et al., 2022). Generally, increasing growth duration promote accumulation of secondary metabolites in perennial plant organs, like root tuber, tuber or rhizome (Kim et al., 2017; Min et al., 2023; Yu et al., 2021; Zhang et al., 2022). In four-year-old *Coptis chinensis* plants, (*S*)-reticuline, a key isoquinoline alkaloid, exhibited significantly higher accumulation compared to two-year-old plants (Min et al., 2023). In *Panax notoginseng*, aged root tissues demonstrate increased accumulation of bioactive saponins (Shi et al., 2007), the total contents of the 11 saponins were 9.82%~14.57% for 2-year-old and 14.20%~16.00% for 3-year-old rhizome (Jia et al., 2013). It was reported that the content of calycosin-7-O-β-D-glucoside in 6-year-old plant was significantly higher than that in 2-year-old of *Astragalus mongholicus* (Zhang et al., 2022). Consequently, extended cultivation period is beneficial to harvest higher-quality Chinese medicinal materials. It holds significant practical values to optimize the harvest time by seeking a balance between time costs and economic benefits (Duan et al., 2023). In its natural habitat, the rhizome of *C. barometz* grows slowly due to its inherent biological traits and environmental constraints. Our year-long study of potted seedlings revealed an annual elongation of merely 1.5-2.0 cm. Although rhizome growth accelerates as the fronds enlarge and produce more carbohydrates via photosynthesis, a minimum cultivation period of 10 years is required to harvest a rhizome measuring 20–30 cm. This extended timeline signifies a markedly slower production cycle for *C. barometz* compared to most agricultural or medicinal species.

While growth retardation limits yield, it remains unknown whether or how the extended growth duration enhances the accumulation of secondary metabolites in *C. barometz* rhizome. To date, the metabolomics profile of *Gou-ji* has not been documented in the literature, and limited knowledge exists regarding its metabolic characteristics (Lim, 2016). It is hypothesized that the prolonged growth duration probably enhances the accumulation of pharmacologically active constituents in the rhizome of *C. barometz*, and that this process is regulated by the coordinated upregulation of key biosynthetic genes. This leads to the central scientific question addressed in this study: “How does the secondary metabolite profile, particularly flavonoid and phenolic acid composition, shift across different developmental stages of the *C. barometz* rhizome, and what are the underlying transcriptomic mechanisms governing these temporal changes?” To investigate these, we sampled rhizome from the same population at three developmental stages: juvenile (YDS), intermediate (MDS), and mature (MS). We performed transcriptomic analysis on fresh rhizome and broadly targeted metabolomics analysis on crude *Gou-ji* materials. Employing integrated multi-omics approaches, we elucidated the secondary metabolite profile and its correlation with growth years, offering insights for enhanced utilization and quality improvement of this traditional Chinese herb.

## Materials and methods

### Plant materials

The Plant *C. barometz* were collected from a natural wild population growing on a forested hillside within the premises of the Shenzhen Orchid Conservation Research Center. To ensure genetic and environmental consistency, all samples were obtained from the same wild population. The age of each plant was estimated by counting the number of leaf scars on the rhizome. For each age group, five individual plants were collected. During sampling, we excised only the fresh rhizome tissue distal to the shoot apical meristem, which corresponded to approximately the distal half of the rhizome. We took care to avoid damaging the leaves and apical meristem so that the remaining portion of the rhizome could continue to grow normally. The collected fresh rhizomes were processed by washing, removing the golden scale, slicing them transversely, and drying them to prepare the *Gou-ji* herbal material.

During slicing, a portion of fresh rhizome from each sample was transferred into a DNase- and RNase-free centrifuge tube, immediately flash-frozen in liquid nitrogen, and stored at -80°C for subsequent molecular and phytochemical analyses.

### Broadly targeted metabolomics assay

Broadly targeted metabolomic profiling of secondary metabolites in *C. barometz* rhizome was performed by the Metware Biotechnology Co., Ltd (Wuhan, China). Pooled rhizome samples were vacuum freeze-dried in a lyophilizer (Scientz-100F; Zhejiang, China) and ground into fine powder using a mill (MM 400; Retsch, Germany). 50 mg tissue powder was homogenized in 1200 μL of a pre-chilled (-20°C) extraction solution (70% methanol/H_2_O, v/v) containing internal standards. The mixture was vortexed (MIX-200; Jingxin, Shanghai, China) for 30 seconds (s) at 30-minute (min) intervals, for six cycles total. Following centrifugation at 12,000 × g for 3 min, the supernatant was collected and filtered through a 0.22 μm nylon membrane syringe filter (Merck Millipore, Germany). The resulting filtrate was transferred to an injection vial for subsequent UPLC-MS/MS analysis.

Sample extracts were analyzed on an ultra-performance liquid chromatography (UPLC) system (ExionLC™ AD, SCIEX, USA) coupled to a QTRAP_®_ 6500+ with mass spectrometry (SCIEX, USA) equipped with an electrospray ionization (ESI) source. Chromatographic separation was performed on an Agilent SB-C18 column (100 mm × 2.1 mm i.d., 1.8 μm; Agilent Technologies, USA) maintained at 40 °C. The mobile phase consisted of solvent A (ultrapure water containing 0.1% formic acid) and solvent B (acetonitrile containing 0.1% formic acid). The following gradient elution program was applied at a flow rate of 0.35 mL/min: 0–9 min, linear gradient from 5% to 95% B; 9–10 min, maintained at 95% B; 10-11.1 min, returned to initial conditions (5% B) and then re-equilibration at 5% B for 2.9 min. The injection volume was 2 μL. The effluent was directly introduced into the ESI source of the triple quadrupole-linear ion trap mass spectrometer. Analysis was carried out in both positive and negative ionization modes with the following source parameters: Ion Spray Voltage, 5500 V (positive ion mode)/-4500 V (negative ion mode); source temperature, 500°C; curtain gas, 25 psi; ion source gas 1 and 2 set at 50 and 60 psi, respectively. The collision-activated dissociation (CAD) was set to a high value.

Metabolite identification was performed by matching secondary mass spectrometry data against the in-house built MetWare database (MWDB). Relative quantification of metabolites was carried out using multiple reaction monitoring mode on a triple quadrupole mass spectrometer. Pearson’s correlation analysis and principal component analysis (PCA) were performed using the MetWare Cloud platform, a free online data analysis tool (https://cloud.metware.cn), to evaluate the correlation between biological replicates within each group and metabolic differences between different groups.

### Transcriptome sequencing and analysis

Total RNA was extracted from the frozen rhizome samples using the RNAprep Pure Plant Plus Kit (Polysaccharides & Polyphenolics-rich) (TIANGEN, China). RNA integrity and concentration were assessed using Qsep400 (Bioptic, China) and Qubit 4.0 Fluorometer (Thermo Fisher Scientific, USA), respectively. Only RNA samples with an RNA Integrity Number (RIN) > 7.0 and sharp ribosomal RNA bands (28S/18S) were used for subsequent library construction. Sequencing libraries were prepared from the qualified RNA samples using a standard protocol, which included mRNA enrichment using oligo(dT) beads, fragmentation, first and second strand cDNA synthesis, adapter ligation, and PCR amplification. The resulting cDNA libraries were quantified by a Qubit fluorometric assay, a fragment analyzer, and quantitative PCR assay. All libraries met the required concentration threshold of > 2 nM for downstream sequencing. The libraries were then sequenced on an Illumina NovaSeq 6000 platform (Illumina Inc, USA).

Raw sequencing reads were processed with Fastp (v0.23.2) under default parameters to remove adapters, poly-N sequences, and low-quality reads, generating high-quality clean reads. Because the alignment rate to the *C. barometz* reference genome (Qin et al., 2024) was low (< 70%), a *de novo* transcriptome assembly approach was adopted. Gene expression levels were estimated by calculating Fragments Per Kilobase of transcript per Million mapped reads (FPKM) values using StringTie (v2.2.1). Differentially expressed genes (DEGs) analysis between groups was performed with the R package DESeq2 (v1.36.0). Genes with an adjusted *p*-value (FDR) < 0.05 and an absolute log2 fold change (|log2FC|) ≥ 1 were considered significantly differentially expressed.

Functional annotation of the assembled transcripts was performed using DIAMOND (v2.1.6) with blastx mode against multiple public databases, including the non-redundant protein (NR) database, SWISS-PROT, TrEMBL. Additionally, the predicted amino acid sequences of unigenes were used to search against the Pfam database using HMMER (v3.3.2) to identify protein domains and families. To functionally characterize the assembled unigenes, Gene Ontology (GO) enrichment and Kyoto Encyclopedia of Genes and Genomes (KEGG) pathway enrichment analyses were performed on the sets of DEGs using the clusterProfiler R package (v4.4.4). Terms and pathways with an FDR-adjusted p-value < 0.05 were considered significantly enriched.

### Total RNA extraction and quantitative real-time PCR

Total RNA was extracted from the frozen rhizome samples using a CTAB-based method modified from Gambino et al. (2010) to accommodate polyphenol-rich plant tissues. The extraction buffer contained 2% cetyltrimethyl ammonium bromide, 2.5% polyvinyl pyrrolidone K30, 2-M NaCl, 100-mM Tris–HCl, 25-mM EDTA-Na_2_, 0.05% spermidine, 2% β-mercaptoethanol. RNA concentration was quantified using a NanoDrop One spectrophotometer (Thermo Fisher Scientific, USA), and integrity was assessed by electrophoresis on a 1% agarose gel. First-strand cDNA was synthesized from 1 μg of total RNA using the PrimeScript™ RT Reagent Kit with genome DNA Eraser (TaKaRa Bio, Japan) according to the manufacturer’s instructions. The first-strand cDNA stock was diluted 20- to 40-fold with nuclease-free water for use in RT-qPCR. RT-qPCR was performed using an ABI QuantStudio 5 Real-Time PCR System (Thermo Fisher, USA) using 96-well plates. Each 14 μL reaction contained 1 μL of diluted cDNA, 7 μL of PerfectStart Green qPCR SuperMix (TransGen Biotech, China), 0.5 μL of each gene-specific primer (10 mmol/L stock solution), and 5 μL of nuclease-free water. The thermal cycling conditions were: initial denaturation at 94°C for 30 s; followed by 40 cycles of denaturation at 94°C for 5 s, and annealing/extension at 60 °C for 30 s. A melting curve analysis was performed to verify the specificity of the amplification.

Based on previous studies evaluating reference gene stability in *C. barometz* (data unpublished), *CbEF1A* was selected as the internal control gene for detecting changes in gene expression abundance in rhizome. Relative gene expression was calculated using the 2^(-ΔΔCt)^ method (Livak and Schmittgen, 2001). The gene-specific primer pairs were synthesized by Sangon Biotech Co., Ltd (Shanghai, China), and the sequences of the primer pairs are listed in **Supplementary Table S1**.

### Quantitative determination of flavonoids

Targeted quantitative metabolomics analysis of flavonoid was performed by MetWare Biotechnology Co., Ltd. (Wuhan, China). The samples were vacuum freeze-dried using a lyophilizer (Scientz-100F, Zhejiang, China) and ground into fine powder using a mill (MM 400, Retsch, Germany). 20 mg of powdered tissue was homogenized in 500 μL of a pre-chilled (-20°C) extraction solution (70% methanol/H_2_O, v/v) containing 10 μL of a mixture of internal standards (4000 nmol/L). The mixture was sonicated for 30 minutes in an ultrasonic bath (KQ5200E; Supmile, China). Following centrifugation at 12,000 *g* for 3 min at 4°C, the supernatant was collected and filtered through a 0.22 μm nylon syringe filter (Merck Millipore, Germany). The resulting filtrate was transferred to an injection vial for subsequent UPLC-MS/MS analysis.

Chromatographic separation was performed on an ACQUITY UPLC HSS T3 C18 (100 mm × 2.1 mm i.d., 1.8 μm, Waters, USA) maintained at 40 °C. The mobile phase consisted of solvent A (ultrapure water containing 0.05% formic acid) and solvent B (acetonitrile containing 0.05% formic acid). The gradient program was as follows: 90% A at 0 min; 80% A at 1.0 min; 30% A at 9.0 min; 5% A at 12.5 min; held at 5% A until 13.5 min; returned to 90% A at 13.6 min; and held at 90% A until 15.0 min. The injection volume was 2 μL. The effluent was directly introduced into the ESI source of the triple quadrupole-linear ion trap mass spectrometer. Analysis was carried out in both positive and negative ionization modes with the following source parameters: ion spray voltage, 5500 V (positive ion mode)/-4500 V (negative ion mode); source temperature, 550 °C; curtain gas, 25 psi; ion source gas 1 and 2 set at 50 and 60 psi, respectively. The collision-activated dissociation (CAD) was set to a high value.

Calibration curves for each target flavonoid were established using a dilution series of standard solutions (0.5, 1, 5, 10, 20, 50, 100, 200, 500, 1000, and 2000 nmol/L). The chromatographic peak areas for the quantitative ion transitions were acquired for each concentration level. Calibration curves were constructed by plotting the peak area ratio (analyte/internal standard) versus the concentration ratio (analyte/internal standard). For analytes without an internal standard, the peak area of the analyte was plotted versus its concentration. The equations for standard curves were listed in **Supplementary Table S2**. The flavonoid content in the samples was calculated using the following formula: X = (c×V×MW)/(10^9^×m) [X: content (μg/g, dry weight); c: concentration values calculated from the standard curve (nmol/L); V: total volume of extraction solution (μL); MW: molecular weight (g/mol); m: sample weight (g)].

## Results

### Secondary metabolite profiling of *Gou-ji* at different developmental stages

**Figure 1:**
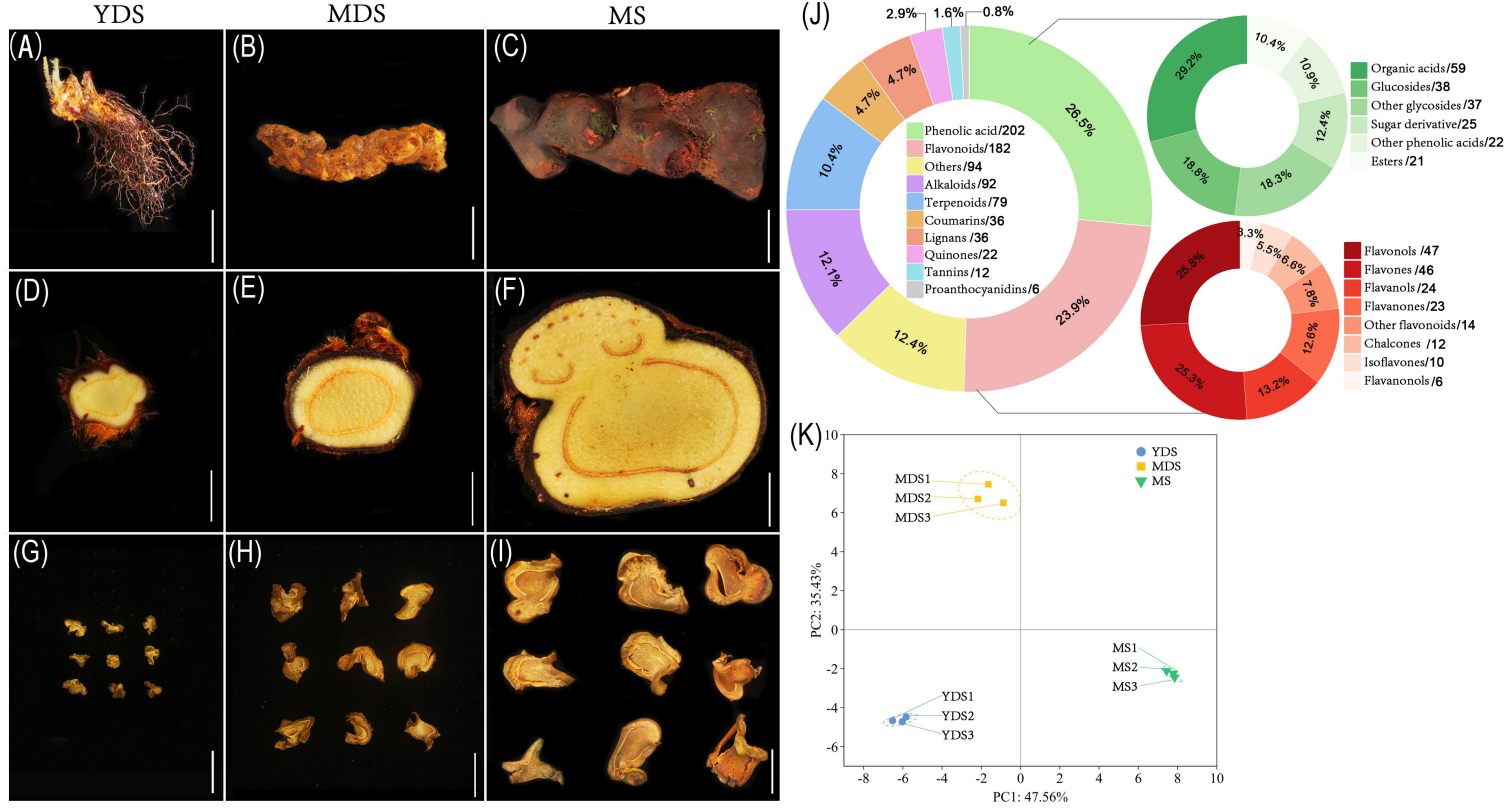
The secondary metabolites in the rhizome of *C. barometz*. The appearance of whole rhizome **(A–C)**, sliced piece of fresh rhizome, and dried *Gou-ji* at different developmental stages: juvenile (YDS, **A, D, G**), intermediate (MDS, **B, E, H**), and mature (MS, **C, F, I**). The white scale bars indicate 2 cm. **(J)** Pie chart depicting class-specific distribution of all secondary metabolites in *Gou-ji*, each number behind forward-slash indicate the count of metabolites. **(K)** Principal component analysis (PCA) of metabolomics data. PC1 (47.56%) and PC2 (35.43%) capture 82.99% of total variance. Circle, square, and inverted triangle represents YDS, MDS, and MS rhizome, respectively. Samples clustered by developmental stage (n=3). Dashed ellipses: 95% confidence regions. YDS, young developmental stage; MDS, medium developmental stage; MS, mature stage.

It is difficult to accurately determine the age of *C. barometz* rhizome based on morphological size (**Figures 1A-I**). The approximate age of the perennial rhizome can be estimated by counting the total number of frond scars, as 2–3 scars formed annually following frond detachment (Chandra, 1970). Hairy scales were observed to shed naturally from older regions of the rhizome (**Figure 1C**), while histological structures remained similar across the three developmental stages (**Figures 1D–F**). The water content of fresh rhizome showed no significant variation among stages, remaining consistent at 77–78% (**Supplementary Figure S1**). This indicates that the rhizome of *C. barometz*, functioning as a sink organ for nutrient transport or storage, exhibited no remarkable alterations in histological structure or water content across different growth years.

The phenolic acid compounds and flavonoids are considered the main bioactive components in natural or processed *Gou-ji*. Therefore, we performed high-throughput secondary metabolite profiling in *Gou-ji* rhizome across three developmental stages using liquid chromatography-mass spectrometry (LC-MS). A total of 761 detectable secondary metabolites were identified in this study (**Supplementary Table S3**), including phenolic acids (202, 26.5%), flavonoids (182, 23.9%), alkaloids (92, 12.1%), terpenoids (79, 10.4%), coumarins (36, 4.7%), lignans (36, 4.7%), quinones (22, 2.9%), tannins (12, 1.6%), proanthocyanidins (6, 0.8%) and others (**Figure 1J**). Together, phenolic acids and flavonoids accounted for over 50% of the total detected secondary metabolites. Phenolic acids were primarily composed of organic acids, glucosides, and sugar derivatives, whereas the flavonoid fraction included flavonols, flavones, flavanols, flavanones, chalcones, isoflavones, and flavanonols. Principal component analysis (PCA) was performed on all metabolomics data to assess the reproducibility among biological replicates. The three biological replicates clustered closely within their respective groups, while samples from distinct developmental stages exhibited clear separation along the principal components (PC1 and PC2) (**Figure 1**). This result demonstrated that the secondary metabolite profiles of *C. barometz* rhizome varied significantly with plant age.

### The secondary metabolites vary in rhizomes at different developmental stages

**Figure 2:**
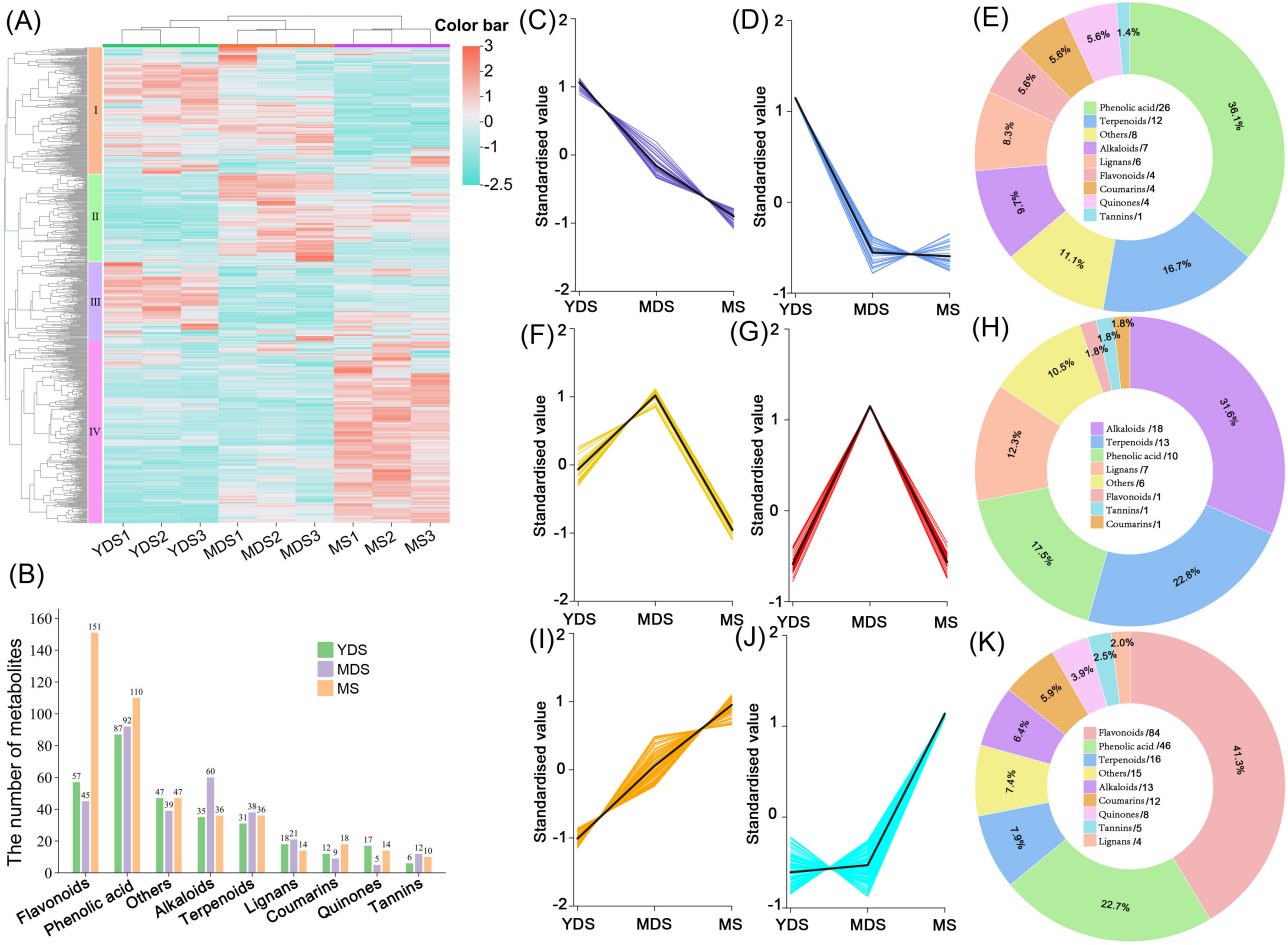
Metabolites analysis of *Gou-ji* from different growth years. **(A)** Clustering heatmap of secondary metabolites. A total of 761 metabolites abundances were normalized by Z-score. Rows: Metabolites; Columns: Samples grouped by developmental stage. Color bar intensities (cyan-white-red: low to high abundance). **(B)** Grouped histogram for the numbers of metabolites belonging to distinct classes. **(C, D, F, G, I, J)** K-mean clustering analysis on differentially abundant metabolites (DAMs, VIP >1, |log_2_FC| >1), 6 groups were clustered with similar dynamic across YDS, MDS and MS rhizomes. **(E, H, K)** Pie chart showing class-specific distribution of enriched metabolites in YDS, MDS, and MS. each number behind forward slash indicate the count of metabolites. YDS, young developmental stage; MDS, medium developmental stage; MS, mature stage.

To elucidate variation in secondary metabolite profiles of *Gou-ji* with different growth years, we performed hierarchical clustering analysis on the secondary metabolomics dataset. Quantified metabolites were standardized by z-score normalization visualized in a heatmap (**Figure 2A**). The two groups, YDS and MDS, formed a cohesive cluster, whereas MS exhibited significant dissimilarity. The heatmap revealed that the metabolic profile of YDS was more closely related to that of MDS, while MS exhibited a distinct metabolic divergence. Metabolites were grouped into four classes based on their enrichment patterns: class I included metabolites co-enriched in MDS and YDS; classes II, III, and IV represented metabolites predominantly accumulated in MDS, YDS, and MS, respectively. Class IV contained the largest number of metabolites, implying that aged (MS) rhizome might accumulate a substantial quantity of secondary metabolites. In terms of metabolite composition, phenolic acids were the most enriched class in YDS rhizome, followed by flavonoids, alkaloids, and terpenoids. Similarly, phenolic acids were predominantly enriched in MDS rhizome, followed by alkaloids, flavonoids, and terpenoids. In contrast, MS rhizome displayed a distinct profile, with flavonoid levels two to three times higher than those in YDS and MDS, followed by phenolic acids, alkaloids, and terpenoids (**Figure 2B**).

We further validated the metabolomics data using orthogonal partial least squares-discriminant analysis (OPLS-DA) and identified differentially accumulated metabolites (DAMs) based on the thresholds of VIP >1 and |log_2_FC| >1. The k-means clustering algorithm was applied to investigate the dynamics of DAMs in *C. barometz* rhizomes across different growth years. Metabolites with coordinated abundance variations were classified into discrete clusters. A total of 72 secondary metabolites were enriched in YDS rhizome, including 26 phenolic acids (**Figures 2C-E**, **Supplementary Table S4**). In MDS rhizome, 57 metabolites were specifically accumulated compared to YDS and MS, with 18 alkaloids showing notably higher abundance at this stage (**Figures 2F–H**, **Supplementary Table S5**). Consistent with the hierarchical clustering results (**Figure 2A**), k-means analysis confirmed significant enrichment of 203 secondary metabolites in MS rhizome (**Figures 2I-K**, **Supplementary Table S6**). Flavonoids constituted the majority (84 metabolites; 41.3% of total), distinguishing MS from the phenolic acid-dominated YDS and alkaloid-enriched MDS profiles. The rhizome of MS exhibited a pronounced enrichment of secondary metabolites, with the quantities of accumulated flavonoids and phenolic acids significantly surpassing those observed in both YDS and MDS. These results demonstrate that metabolic dynamics in *C. barometz* rhizome were strongly correlated with growth year, showing a progressive enrichment of secondary metabolites—particularly flavonoids—with increasing age.

### The flavonoids especially enrich in rhizome at maturation stage

**Figure 3:**
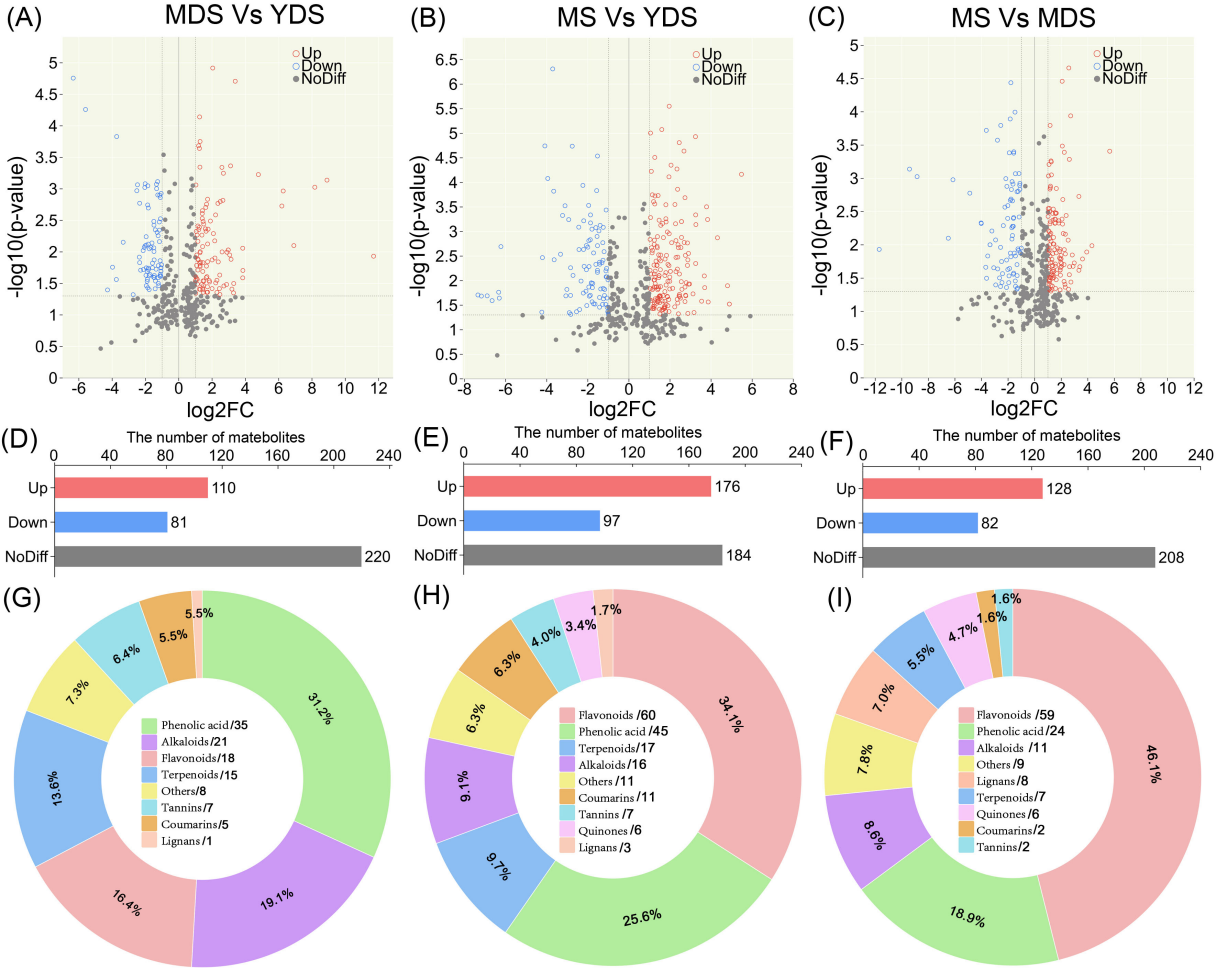
Pairwise comparisons of metabolomic differences among rhizomes with different growth years. **(A–C)** Volcano plot illustrating DAMs in distinct pairwise comparisons. Each circle represents a quantified metabolite. The horizontal dashed line marks the significance threshold (-log_10_(*p*-value) = 1.3, *p*-value < 0.05). Vertical dashed lines indicate a |log_2_(FC)| > 1 threshold (FC > 2). Red and blue circles denote significantly enhanced and reduced metabolites, respectively; gray points represent metabolites with non-significant changes. **(D–F)** Histogram for the number of metabolites in pairwise comparisons. **(G–I)** Pie chart presenting class-specific distribution of enriched metabolites in pairwise comparisons, ach number behind forward-slash indicate the count of metabolites. YDS, young developmental stage; MDS, medium developmental stage; MS, mature stage.

To investigate the dynamic changes in secondary metabolites, particularly for flavonoids and phenolic acids, in relation to the growth years of *C. barometz* rhizome, we performed pairwise metabolomic comparisons across growth stages (**Supplementary Table S7**). Volcano plot analysis of the MDS vs. YDS comparison revealed 110 secondary metabolites significantly enriched in MDS rhizome which have a longer growth duration (**Figures 3A, D**). Among these metabolites, phenolic acids were the most abundant class, followed by alkaloids, flavonoids, and terpenoids (**Figure 3G**). In the MS vs YDS comparison, a greater number of DAMs accumulated in MS rhizome, where flavonoids represented the most significantly enriched class, ahead of phenolic acids, terpenoids, and alkaloids (**Figures 3B, E, H**). Distinct metabolic dynamics were discovered in the aged rhizomes (MDS and MS), as evidenced by their differential metabolite profiles compared to the YDS rhizome. A quantitative metabolomics comparison between the two aged rhizomes, MS and MDS, identified 128 DAMs (Fold Change > 2, *p* < 0.05), with flavonoid abundances (59) almost matching those from the MS/YDS comparison (60) (**Figures 3C, F, I**). Pie chart analysis further confirmed that flavonoids and phenolic acids collectively accounted over 50% of the DAMs in MS rhizome (**Figures 3H, I**). This finding is consistent with the established pharmacology of *Gou-ji*, in which these two classes were considered major bioactive components. Together, these findings demonstrate a clear developmental trajectory in secondary metabolite composition with prolonged growth.

**Figure 4:**
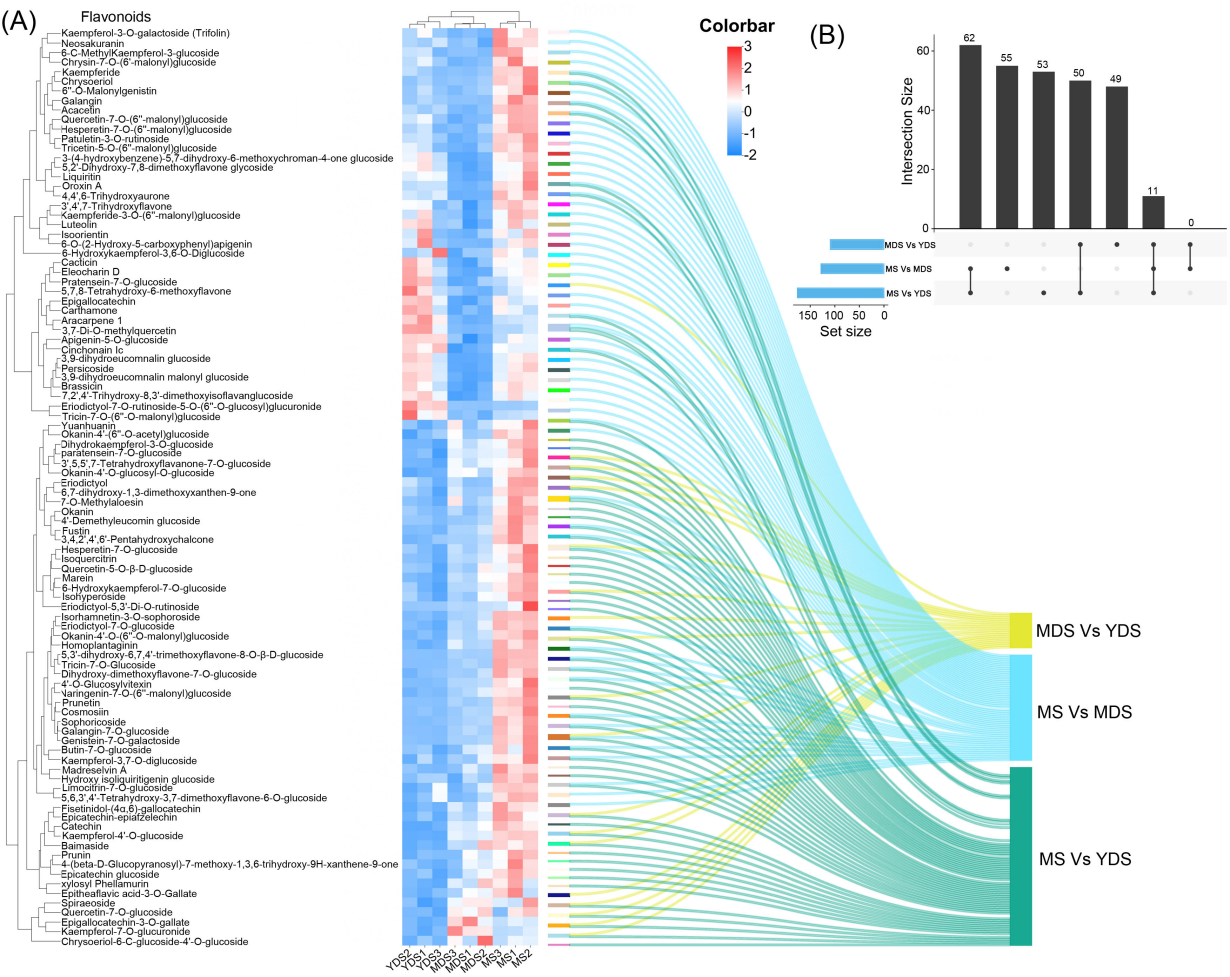
Comparison of flavonoids enrichment among three developmental stages. **(A)** Clustering heatmap for the differentially abundant flavonoids (DAFs) in the pairwise comparisons. Rows: DAFs; Columns: Samples grouped by developmental stage. Color bar intensities (blue-white-red: low to high abundance). Sankey diagram showing the correspondence between significantly DAFs and their enrichments in MDS or MS rhizome. **(B)** An upset plot depicting the intersection of DAFs lists from three comparative analyses (MDS vs YDS, MS vs MDS, and MS vs YDS.). The top black bars indicate the size of the intersection for each specific combination of sets directly below it. The bottom blue bars represent the size of individual set. YDS, young developmental stage; MDS, medium developmental stage; MS, mature stage.

Metabolomics profiling identified phenolic acids as the most abundant class of secondary metabolites in *C. barometz* rhizome through metabolomics profiling (**Figure 1J**). Among all DAMs, phenolic acids exhibited the most significant dynamic fluctuations in MDS compared to YDS (**Figure 3G**, **Supplementary Figure S2A**), and they were also the most numerous among metabolites that decreased significantly in both the MS/YDS and MS/MDS comparisons (**Supplementary Figures S2B, C**). Venn diagrams illustrated overlaps and unique distributions of DAMs across comparison groups. The MDS/YDS, MS/YDS and MS/MDS groups contained 49, 53 and 55 unique DAMs, respectively, with11 common to all three. A total of 62 DAMs overlapped between MS/YDS and MS/MDS, and 50 between MDS/YDS and MS/YDS. By contrast, no DAMs were shared between MS/MDS and MDS/YDS (**Figure 4B**, **Supplementary Table S8**), indicating systematic differences in metabolite profiles across developmental stages and highlighting substantial metabolic restructuring over time. The high overlap of DAMs between the MS/YDS and MS/MDS comparisons indicated that MS rhizome underwent continuous metabolite accumulation relative to both YDS and MDS rhizomes. Upon maturation (MS stage), the rhizome maintains a relatively stable yet highly enriched metabolite composition, suggesting a functional transition toward a specialized storage organ. The results clearly demonstrated that the metabolite compositions among the groups exhibit both shared commonalities and distinct characteristics, reflecting the divergent yet interconnected nature of plant metabolic pathways under different growth year conditions.

Flavonoids were preferentially enriched in aged rhizome, with the most substantial accumulation observed in the MS stage (**Figures 3H, I**). A clustered heatmap of differentially abundant flavonoids (DAFs) visualized their marked accumulation in MS compared to YDS and MDS. Their abundance was substantially higher than that in both YDS and MDS (**Figure 4A**). In detail, the MDS/YDS, MS/YDS and MS/MDS comparisons contained 4, 21 and 32 unique DAFs, respectively, with 2 common to all three groups. A total of 25 DAFs overlapped between MS/YDS and MS/MDS, and 12 between MDS/YDS and MS/YDS. Again, no DAFs were common to the MS/MDS and MDS/YDS groups (**Figure 4A**, **Supplementary Figure S3**, **Supplementary Table S9**). The growth period from MDS to MS likely represents a critical phase for the accelerated accumulation of flavonoids accumulation in the rhizome of *C. barometz*. This was supported by the high number of shared DAFs (25) between the MS/YDS and MS/MDS comparisons, which was greater than the number shared between the MDS/YDS and MS/YDS comparisons (12). In the MS/YDS comparison, flavones (13 compounds) were the predominant DAFs in MS rhizome, followed by flavanones (12), flavonols (12), chalcones (8), flavanols (6), and isoflavones (5). Similarly, in the MS/MDS comparison, flavones (19) constituted the predominant category of DAFs, followed in descending abundance order by flavonols (14), isoflavones (6), flavanones (6), and chalcones (4). These differential enrichment pattern underscores stage-specific biosynthetic regulation, with the aged rhizome phase (MS) demonstrating enhanced metabolic coordination for flavonoid production.

### The genes involved in flavonoids biosynthesis differentially expressed in rhizome at maturation stage

To elucidate the molecular mechanisms underlying flavonoid enrichment in mature rhizome, we performed transcriptome sequencing of rhizome of varying growth years. We then systematically screened for differentially expressed genes (DEGs) to obtain high-throughput gene expression profiles. The dimensionality reduction analysis revealed PC1 (58.78% variance) and PC2 (25.04% variance) as the dominant components, collectively explaining 83.82% of the transcriptional variation (**Supplementary Figure S4**). The clear separation of sample clusters along the PC1 and PC2 axes demonstrated statistically significant transcriptomic heterogeneity among the groups. DEG identification (|log_2_(fold change)| > 1, p < 0.05) yielded 15,764 genes in the MDS vs YDS comparison, including 6,668 upregulated and 9,096 downregulated genes (**Supplementary Figures S5A, B**). This predominant downregulation suggests transcriptional suppression during the transition from YDS to MDS. In contrast, comparisons involving the MS stage revealed more extensive transcriptional changes: the MS vs YDS group contained 25,552 DEGs (13,083 upregulated, 12,469 downregulated; **Supplementary Figures S5C, D**), and the MS vs MDS group contained 20,873 DEGs (11,804 upregulated, 9,069 downregulated; **Supplementary Figures S5E, F**). Transcriptional profiling revealed that the MS rhizome displayed enhanced transcriptional activity, with a predominance of upregulated DEGs. The number of DEGs increased with the developmental divergence between compared stages. Notably, the number of upregulated genes was significantly greater than that of downregulated genes in comparisons involving the MS stage.

Venn analysis identified 10,503 DEGs common to both the MS vs. MDS and MS vs. YDS comparisons. Notably, among these shared DEGs, 66.7% (7,005) were upregulated and 33.2% (3,489) were downregulated (**Supplementary Figure S6**). Among 7,429 DEGs common to MDS vs YDS and MS vs YDS, a reverse pattern was observed: 29.1% (2,162) were upregulated and 70.9% (5,267) downregulated. Unique DEGs numbered 2,171 in MDS vs YDS, 4,206 in MS vs YDS, and 5,389 in MS vs MDS, with a notable upregulation bias in MS-related comparisons (**Supplementary Figure S6**). Given the established role of transcription factors (TFs) in regulating flavonoid biosynthesis (Liu et al., 2021), we analyzed TF expression and identified significant enrichment of MYB, WRKY, bHLH, bZIP, NAC, and ERF family members in MS rhizome. Specifically, 188 TFs were upregulated in MS vs YDS, and 190 in MS vs MDS, with a core set of 120 TFs commonly upregulated in both comparisons.

**Figure 5:**
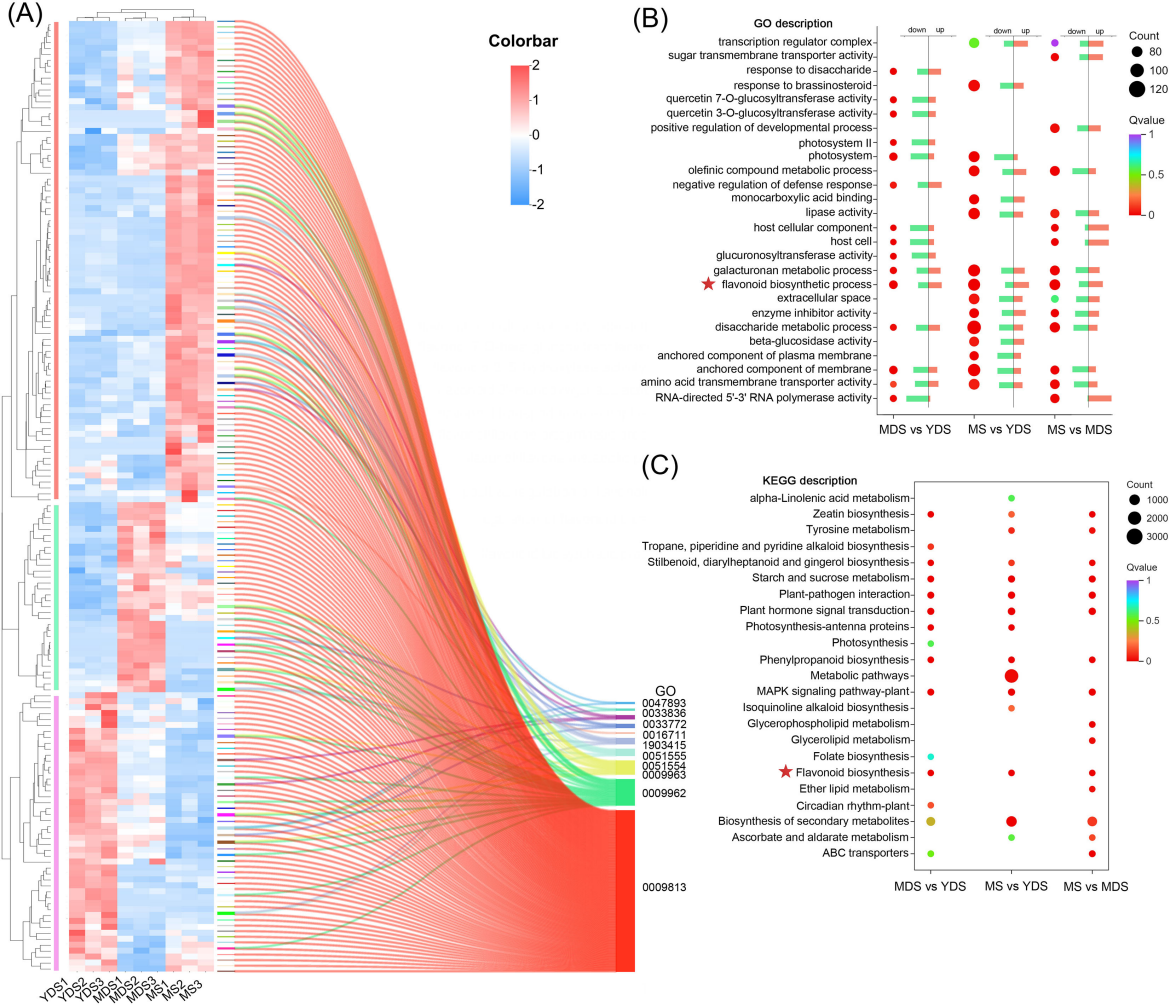
The transcriptome data analysis of rhizomes from different growth years. **(A)** clustering heatmap of DEGs involved in flavonoid biosynthesis pathway. Rows: DEGs; Columns: Samples grouped by developmental stage. Color bar intensities (blue-white-red: low to high abundance). The clustering tree was delineated into three subclasses using vertical bars colored in three distinct colors (red, green, and purple). Sankey diagram presents the correspondence between significant DEGs and their associated GO terms. All listed GO terms are closely associated with the flavonoid biosynthesis pathway. **(B)** GO enrichment analysis of DEGs for biological Process. The dot plot displays the top 15 enriched GO terms for each pairwise comparison. The bar charts show the percentage of up-regulated and down-regulated DEGs in each GO term. **(C)** The dot plot displays the top 15 enriched KEGG pathway for each pairwise comparison. Dot size reflects the count of DEGs, and color bar represents the -log10(p-adjust) value. YDS, young developmental stage; MDS, medium developmental stage; MS, mature stage.

The Gene Ontology (GO) database was used for functional enrichment analysis and annotation of DEGs in distinct comparative groups. The bubble plot displayed the top 5 enriched GO terms in each of the three categories: biological processes (BP), cellular components (CC), and molecular functions (MF) (**Figure 5B**). The relative percentages of up- and down-regulated DEGs within each GO term were visualized using bar charts (**Figure 5B**). The significantly enriched GO terms (Q value < 0.05) varied substantially across experimental groups, as did the number of DEGs assigned to each term. Notably, the MS vs. YDS group had the most DEGs (91–125 DEGs/term) associated with these GO terms, suggesting significant transcriptional reprogramming at this stage. In contrast, the MDS vs. YDS group showed fewer DEGs (63–81 DEGs/term) assigned to individual GO terms, suggesting limited divergence in transcriptional dynamics between these two stages. Shared enriched terms (Q < 0.05) across comparisons included flavonoid biosynthetic process (GO:0009813, BP), disaccharide metabolic process (GO:0005984, BP), galacturonan metabolic process (GO:0010393, BP), anchored component of membrane (GO:0031225, CC), and amino acid transmembrane transporter activity (GO:0015171, MF). In detail, the flavonoid biosynthetic process was enriched for 81, 111 and 100 DEGs in MDS vs. YDS, MS vs. YDS and MS vs. MDS comparisons, respectively. Notably, a significant proportion of these DEGs exhibited marked upregulation in elder rhizome (MS) compared to younger (MDS or YDS) stages (**Figure 5A**).

We further identified 160 DEGs associated with 10 flavonoid-related GO terms (**Figure 5A**, **Supplementary Tables S10**, **S11**). The heatmap displayed the transcriptional dynamics of these DEGs across developmental stages in the rhizome. YDS and MDS samples clustered together on one branch, which was distinct from the MS cluster, indicating that the transcriptional profiles of these flavonoid-related DEGs in MS rhizome were markedly different. The vertical hierarchical clustering heatmap revealed three principal dendrogram branches that corresponded to the three developmental stages, where rhizome at the maturation stage (MS) had the largest number (81 of 160) of DEGs with elevated expression levels. Enrichment analysis revealed higher Rich Factor values for nine of the ten flavonoid-related GO terms in both the MS vs. YDS and MS vs. MDS comparisons than in the MDS vs. YDS comparison, supporting enhanced flavonoid pathway activity at maturity (**Supplementary Figure S7**, **Supplementary Table S12**).

KEGG pathway analysis highlighted the top 15 enriched pathways, categorized into metabolism, environmental information processing, and organismal systems (**Figure 5C**). The majority of enriched KEGG pathways across the three comparison groups were associated with metabolic pathways, indicating that a predominant portion of DEGs were functionally involved in metabolic processes within rhizome. Multiple KEGG pathways associated with plant flavonoid biosynthesis were identified, including secondary metabolite biosynthesis (ko01110), flavonoid biosynthesis (ko00941), and phenylpropanoid biosynthesis (ko00940). The MS vs YDS group showed the highest DEG enrichment in the “Metabolic pathways” (ko01100) category. Integrated transcriptomic and functional analyses thus demonstrate that unique metabolic reprogramming patterns in rhizomes at different developmental stages. Transcriptomic profiling revealed that flavonoid accumulation in MS rhizome was mechanistically linked to temporal expression dynamics of genes associated with secondary metabolism, particularly those involved in plant flavonoid biosynthesis pathways.

### The transcriptional dynamic of flavonoid-related genes in developmental rhizome

**Figure 6:**
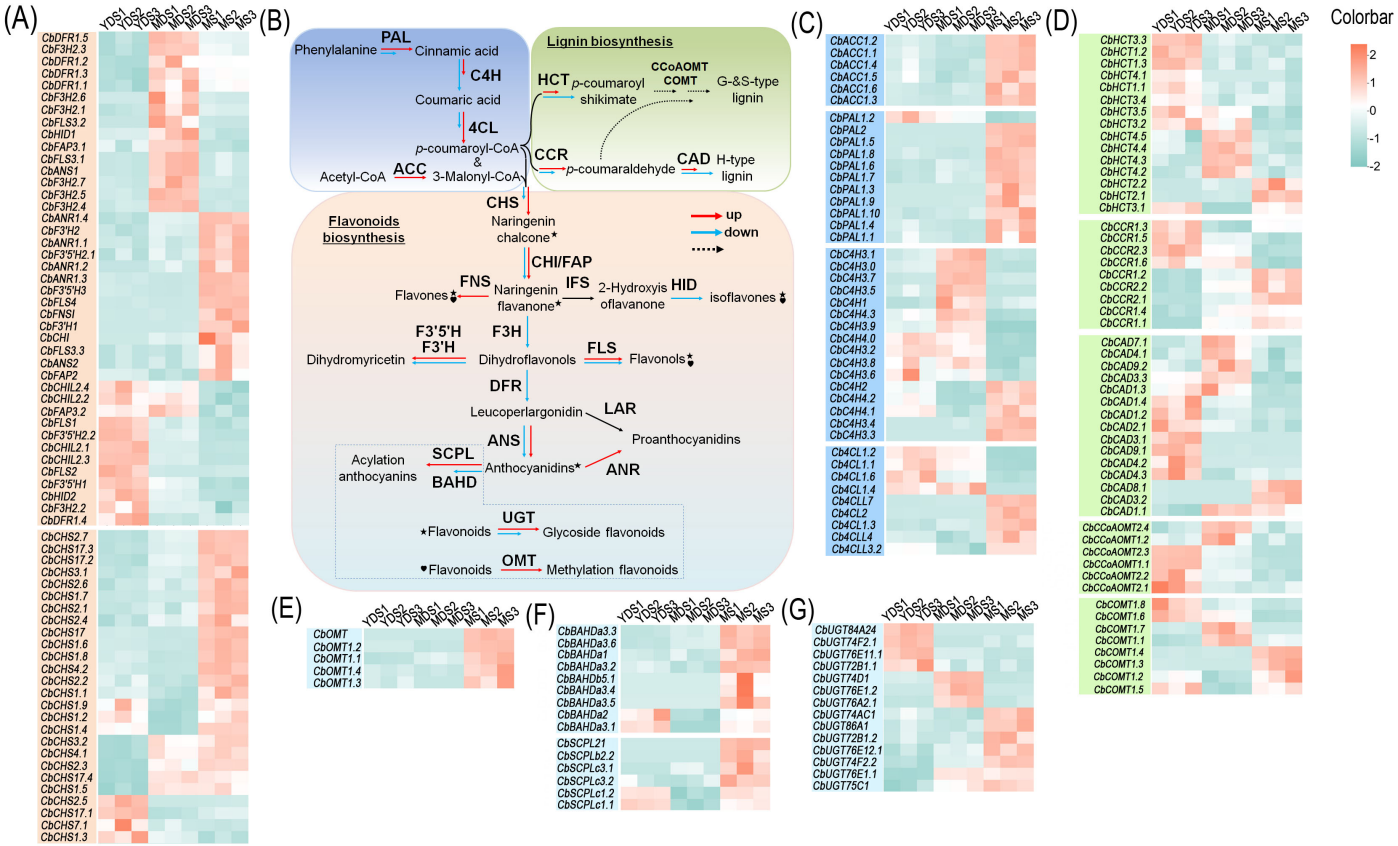
Expression abundance of key enzymatic genes in the flavonoid biosynthetic pathway. **(A)** Enzyme-coding genes involved in flavonoid biosynthesis. **(B)** A brief schematic for flavonoid and lignin biosynthetic pathway. Solid arrows represent direct enzymatic steps, while dashed arrows indicate multi-step reactions. Red arrows indicate up-regulated genes, and blue arrows for down-regulated genes. Specific modification steps are highlighted with symbols: star for glycosylation and heart for methylation. **(C)** Enzyme-coding genes involved in precursor preparation. **(D)** Enzyme-coding genes involved in lignin biosynthesis. **(E-G)** Enzyme-coding genes involved in producing flavonoid derivatives. Color bar intensities (cyan-white-red: low to high abundance). YDS, young developmental stage; MDS, medium developmental stage; MS, mature stage.

Flavonoids play essential biological roles in plants, and their biosynthetic pathways—along with the associated enzymatic and regulatory genes—have been extensively characterized (Liu et al., 2021). Based on gene function databases and transcriptome data from *C. barometz* rhizome, we identified multiple structural genes involved in flavonoid biosynthesis (**Figures 6A, C**). These enzyme-encoding genes are depicted in a schematic of the flavonoid biosynthetic pathway (**Figure 6B**), which is well established (Liu et al., 2021). Enzyme-encoding genes, including phenylalanine ammonia-lyase (*PAL*), cinnamic acid 4-hydroxylase (*C4H*), 4-coumarate-CoA ligase (*4CL*), and acetyl-CoA carboxylase (*ACC*), catalyze the initial substrate preparation for flavonoid biosynthesis. Transcriptomic analysis revealed constitutive expression of these genes and their homologs in *C. barometz* rhizomes during juvenile, intermediate, and mature stages (**Figure 6C**). Some genes exhibited significantly higher expression levels in mature rhizome compared to the other two stages, suggesting stage-specific roles in flavonoid biosynthesis. We annotated 26 putative *CHS* genes in the transcriptome, 22 of which were upregulated in mature rhizome (**Figure 6A**), suggesting enhanced production of naringenin chalcone—the core scaffold for diverse flavonoids such as flavones, chalcones, flavonols, isoflavones, and flavanols (Liu et al., 2021). We also annotated numerous enzyme genes and their homologs that catalyze the conversion of naringenin chalcone into diverse flavonoids (**Figure 6A**). These genes exhibited ubiquitous expression in rhizomes at different developmental stages, with some showing significant upregulation in mature rhizome, including *CbCHI*, *CbFLS*, *CbANR*, *CbF3’5’H*, *CbF3’H*, and *CbFAP* (**Figure 6A**). To validate the reliability of the transcriptomic data, we examined the expression levels of several CHS genes specifically expressed in mature rhizome using qRT-PCR technology. With the exception of a few CHS genes that were undetectable, the expression trends of the remaining CHS genes were largely consistent with the transcriptomic results (**Supplementary Figure S8**).

The predominant flavonoids accumulated in the MS rhizome were glycosides, methoxyisoflavones, and malonylated derivatives (**Supplementary Figure S4**). These substituents were bound to various positions on the C6-C3-C6 core skeleton, governing the stability and biological properties of the flavonoids (Shen et al., 2022). Transcriptome analysis identified numerous enzyme-encoding genes potentially involved in flavonoid methylation, acylation, and glycosylation. Expression heatmaps revealed significantly elevated transcript abundances for many of these genes in mature rhizome compared to the other two developmental stages (**Figures 6E–G**). This elevated expression likely promotes the biosynthesis of stabilized flavonoid derivatives during maturation.

In plants, *p*-coumaroyl CoA, an intermediate in the phenylpropanoid metabolic pathway, serves not only as a pivotal substrate for flavonoid biosynthesis but also as a key substrate for lignin monomer synthesis (Vogt, 2010). We identified key enzyme genes from the lignin biosynthesis pathway in the transcriptome, including cinnamoyl-CoA reductase (CCR), hydroxycinnamoyl-CoA hydroxycinnamoyl transferase (HCT), caffeoyl-CoA O-methyltransferase (CCoAOMT), caffeic acid O-methyltransferase (COMT) and cinnamyl alcohol dehydrogenase (CAD) were identified from the transcriptome. The heatmap revealed higher transcript levels of these genes and their homologs in juvenile rhizome (**Figure 6D**), likely because juvenile rhizome required more lignin monomers to complete morphogenesis. In contrast, most of these genes showed low expression in mature rhizome, with the exception of CCR and its homologs.

### Quantitative analysis of flavonoid contents in the rhizome of *C. barometz*

**Figure 7:**
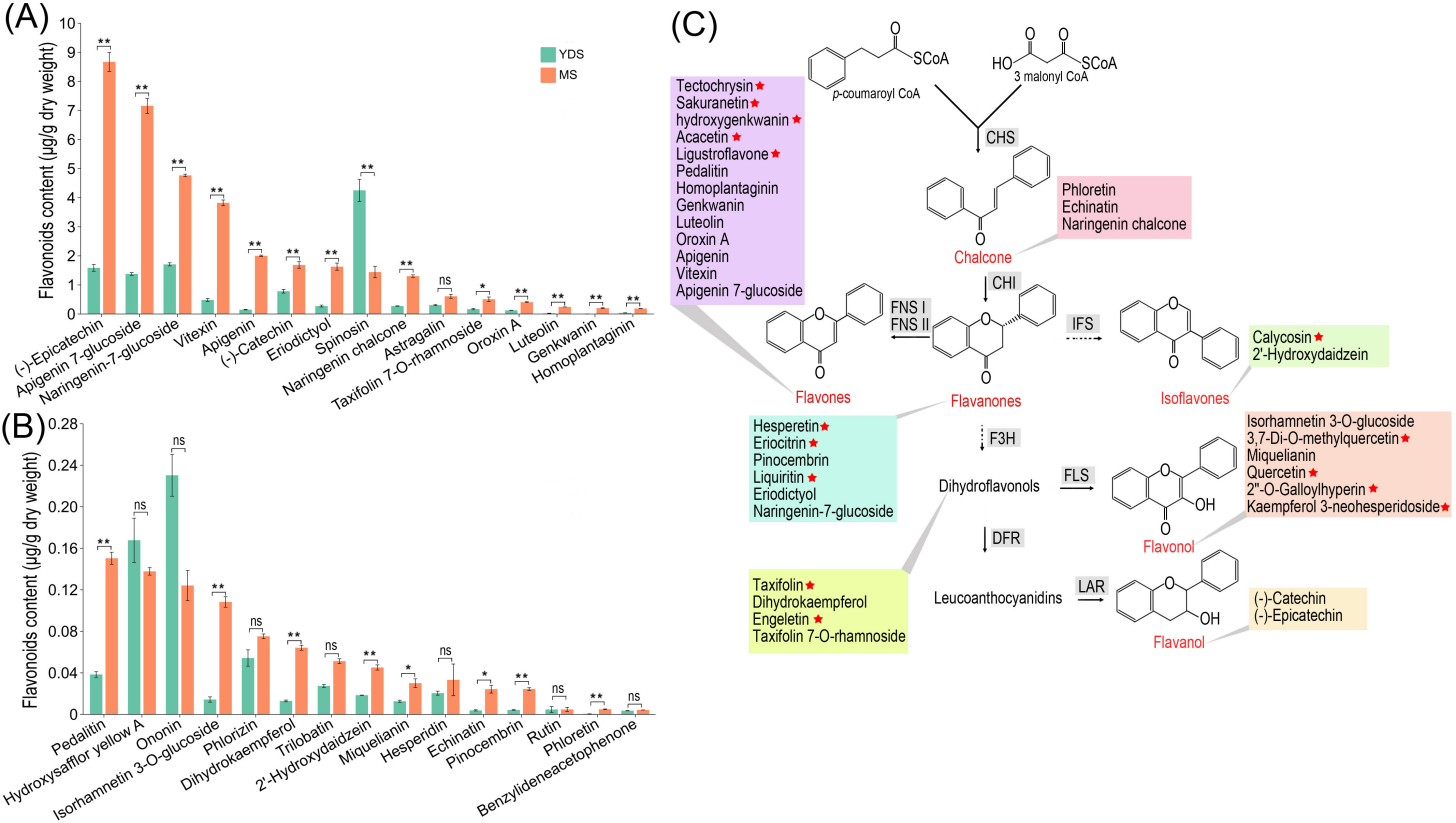
Flavonoid contents in the rhizome of *C*. *barometz*. **(A, B)** The contents of 30 flavonoids in YDS and MS rhizome. Asterisks in a and b indicate statistically significant differences: *, *p* < 0.05, **, *p* < 0.01. ns means no significance. **(C)** A brief schematic diagram for flavonoid biosynthesis. 36 flavonoid compounds are categorized into 7 subclasses. 15 compounds unique to MS rhizomes are marked with a red star. YDS, young developmental stage; MS, mature stage.

To further investigate flavonoid content in *C. barometz* rhizomes from different growth years, we used ultra-performance liquid chromatography coupled with tandem mass spectrometry (UPLC-MS/MS) to quantify 185 flavonoids in both YDS and MS rhizomes (**Supplementary Table S13**). We detected 48 flavonoid compounds in total, with 45 quantified in MS and 33 in YDS rhizome (**Supplementary Figure S9**). Comparative analysis revealed that 30 flavonoids were common to both types, while 15 and 3 were exclusive to MS and YDS rhizomes, respectively. Applying criteria of fold change (FC > 2) and false discovery rate (FDR < 0.05), we found that among the 30 common flavonoids, 21 were significantly more abundant in MS than in YDS rhizome, and only one was significantly less abundant (**Figures 7A, B**). Absolute concentrations indicated that most flavonoids were present at low levels (< 1.0 μg/g). In MS rhizome, nine compounds exceeded this threshold: (-)-epicatechin, apigenin-7-glucoside, naringenin-7-glucoside, vitexin, apigenin, (-)-catechin, eriodictyol, spinosin, and naringenin chalcone. In contrast, only four flavonoids, (-)-epicatechin, apigenin-7-glucoside, naringenin-7-glucoside, and spinosin, were detected at concentrations >1.0 μg/g in YDS rhizome. The 36 flavonoids that were either specific to MS or more abundant there than in YDS were primarily flavones (13/36), followed by flavonols, flavanones, dihydroflavonols, chalcones, isoflavones, and flavanols (**Figure 7C**). Overall, MS rhizome exhibited enhanced flavonoid biosynthetic capacity throughout the pathway compared to YDS rhizome, consistent with metabolomic profiling data (**Figure 3H**).

## Discussion

### Progressive flavonoid enrichment during rhizome maturation of *C. barometz*

*Gou-ji* is a widely used traditional Chinese medicine commonly employed to treat age-related joint pain and lumbar-knee debility in elderly populations (Chen et al., 2022; Huang et al., 2018; Huang et al., 2019; Liu et al., 2020; Zhao et al., 2011). The ‘*Chinese Pharmacopoeia’* (2020 edition) established protocatechuic acid (3,4-dihydroxybenzoic acid; C_7_H_6_O_4_) as the exclusive chemical quality marker for its medicinal rhizome, stipulating a minimum content threshold of 0.02% by dry weight. However, wild-collected *C. barometz* rhizomes of different ages are often pooled, minimally processed (cleaning and slicing), and then directly used as *Gou-ji* in commerce. Rhizomes of different growth years are often not systematically classified, and quantitative analysis of marker compounds is rarely performed. To date, a systematic characterization of the phytochemical profiles in rhizomes across different growth years is lacking in the scientific literature (Lim, 2016). Here, we employed broadly targeted metabolomics to characterize the secondary metabolites in rhizomes across three developmental stages. Comparative metabolomic analysis further delineated stage-specific secondary metabolite signatures that exhibited temporal correlations with prolonged growth duration. Comprehensive metabolomic profiling revealed progressive flavonoid enrichment during rhizome maturation. Concurrently, transcriptomic analysis identified a significant upregulation of flavonoid biosynthetic genes in MS rhizome, suggesting a molecular basis for this accumulation. Our broadly targeted quantitative profiling identified 36 flavonoids that were significantly enriched in MS rhizome, further substantiating that prolonged growth duration enhances flavonoid accumulation in *C. barometz*.

In perennial medicinal plants analogous to *C. barometz*, the accumulation of pharmacologically active constituents often exhibits dynamic changes commonly correlated with the duration of growth. This age-dependent metabolite accumulation phenomenon has been observed in various traditional Chinese medicines (Hazrati et al., 2024). It is well-established that a prolonged harvest period facilitates the accumulation of bioactive compounds in perennial organs, including ginsenosides in *P. ginseng* (Shi et al., 2007), (S)-reticuline in *C. chinensis* (Min et al., 2023), saponins in *P. notoginseng* (Jia et al., 2013; Yan et al., 2024), calycosin in *A. mongholicus* (Zhang et al., 2022), bioactive sesquiterpenoids and phenolic acids in *Atractylodes macrocephala* (Cui et al., 2025). Nuts from centennial and millennial *T. grandis* trees showed a marked increase in total flavonoid content compared to those from 10-year-old trees (Yan et al., 2023). Indeed, the association between growth duration and bioactive constituent accumulation does not invariably represent a strict positive correlation. A case in point is the study by Yuan et al. (2022) on the stem of *Dendrobium. moniliforme*, which clearly demonstrated that while flavonoids such as chalcone, naringenin, eriodictyol, dihydroquercetin, and other flavonoids accumulated substantially in the third year, this trend did not continue into the fourth year. Parallel findings were reported by Wang et al. (2022) for the leaf of *Ginkgo biloba*, demonstrating a marked decrease in flavonoid content with increasing age in 1- to 7-year-old trees. Perennial fleshy roots, tuberous roots, and rhizome function primarily as storage organs, in which the levels of bioactive constituents are progressively enriched with increasing growth duration. In contrast, the content of active chemical components in photosynthetic organs such as leaves and stems exhibits dynamic variations in response to developmental stage and environmental conditions.

### Chemodiversity of *Gou-ji*: phenolic acids and flavonoids as predominant phytochemistry classes

Unlike other medicinal plants (e.g., *Huperzia* sp*ecies* with huperzine A, or Taxus species with paclitaxel), no single, dominant bioactive compound has been characterized for *Gou-ji*. Medicinal research on *Gou-ji* has extensively focused on the isolation and characterization of its phytochemical constituents. Numerous secondary metabolites have been characterized from the raw and processed rhizome, including pterosins, ptaquiloside, sterols, saccharides, phenolic acids, flavonoids, alkaloids and terpenoids (Lim, 2016). The total phenolic acid content in *C. barometz* rhizome sourced from various regions across China ranged from 3.72% to 6.16%, while in processed products, it ranged from 3.09% to 5.09% (Ju et al., 2012). This study identified phenolic acids and flavonoids as the predominant secondary metabolites in *C. barometz*, collectively accounting for over half of all detected metabolites. Phenolic acids and flavonoids, which are prominent plant secondary metabolites, possess significant biological activities, including antioxidant, anti-inflammatory, and antibacterial effects (Freitas et al., 2024; Kiokias et al., 2020; Liu et al., 2021; Shen et al., 2022). The pharmacological effects of *Gou-ji* in traditional Chinese medicine may be attributed primarily to these bioactive constituents (Lim, 2016). Although phenolic acids and flavonoids constitute the predominant secondary metabolite classes in *C. barometz* rhizome, their specific enrichment profiles shift dynamically during development. In perennial plant organs, chronological age governs both internal tissue architecture and the cellular repertoire of accumulated secondary metabolites (Cui et al., 2025; Yan et al., 2024; Wang et al., 2022; Yuan et al., 2022). As demonstrated by the metabolomic data in this study, the rhizome at the YDS, MDS and MS predominantly accumulate phenolic acids, alkaloids, and flavonoids, respectively.

### Flavonoids might be key bioactive constituents in mature rhizome of *C. barometz*

Prior functional analyses of *Gou-ji*’s phytochemical constituents have not adequately investigated the bioactivity of flavonoids (Lim, 2016). Our high-throughput omics data revealed significant enrichment of flavonoid in MS rhizome of *C. barometz* compared to the MDS and YDS. Given their well-documented multifaceted bioactivities (Wang et al., 2018), flavonoids are likely primary drivers of *Gou-ji*’s pharmacological properties, a role that has been previously undercharacterized. Quantitative analysis by UPLC-MS/MS identified numerous flavonoids in MS rhizome. High levels were detected for (-)-epicatechin (8.67 μg/g), apigenin-7-glucoside (7.16 μg/g), naringenin-7-glucoside (4.77 μg/g), vitexin (3.82 μg/g), apigenin (2.00 μg/g), (-)-catechin (1.69 μg/g), eriodictyol (1.63 μg/g), spinosin (1.44 μg/g), and naringenin chalcone (1.30 μg/g). These compounds and their derivatives, which are widely distributed in vascular plants, have well-documented bioactive properties, particularly their therapeutic potential (Baranwal et al., 2022; Felgines et al., 2000; Fraga et al., 2018; Salehi et al., 2019). Collectively, these findings establish a foundation for elucidating the pharmacological activities of flavonoids in *Gou-ji*, highlighting them as key bioactive constituents.

Currently, the *Chinese Pharmacopoeia* designates protocatechuic acid as the sole chemical marker for the quality assessment of *Gou-ji*. While this single-marker approach has served a role in basic quality control, our findings suggest that it may not comprehensively reflect the medicinal value of this herb. Our study revealed significant enrichment of flavonoids in the mature rhizome of *C. barometz*, including compounds such as (-)-epicatechin, apigenin-7-glucoside, and vitexin, which are known for their notable antioxidant, anti-inflammatory, and potential bone-strengthening activities. These specific flavonoids are likely integral to the traditional efficacy of *Gou-ji* in “strengthening bones and muscles” and “invigorating the lumbar and knee regions.” From a practical perspective, establishing a multi-component quality control system centered on flavonoids represents a promising direction for standardizing *Gou-ji*. Two practical implementation strategies are available: determination of total flavonoid content or quantification of key characteristic flavonoids.

### Carbon partitioning at the p-coumaroyl-CoA node: balancing lignin and flavonoid biosynthesis

As mentioned previously, *p*-coumaroyl-CoA represents a critical branch point precursor in plant secondary metabolism, channeling carbon flux toward the biosynthesis of distinct compound classes, including flavonoids and lignin (Vogt, 2010). Consequently, the biosynthesis of plant flavonoids and lignin involves substrate competition at the p-coumaroyl-CoA node (Wang et al., 2024). Studies have demonstrated that flavonoid accumulation can inhibit lignin biosynthesis in plant cells, thereby impacting growth (Besseau et al., 2007). Lignin, an integral structural component of vascular tissues, plays a major role in plant morphological development. Conversely, flavonoids function as pivotal stress-responsive metabolites that mediate adaptation to environmental pressures. This biosynthetic crosstalk thus represents a core mechanism balancing resource allocation between developmental growth and stress resistance (Yang et al., 2025). Comparative transcriptome analysis indicated enhanced expression of lignin biosynthetic pathway genes in YDS rhizome compared to MS rhizome. For instance, HCT is a key catalytic enzyme that converts p-coumaroyl-CoA to p-coumaroyl shikimate in plant lignin biosynthesis. Our transcriptomic analysis identified 15 HCT genes, with 12 being significantly downregulated in MS rhizome compared to YDS and MDS, suggesting suppressed lignin biosynthesis upon rhizome maturation. Concomitantly, we quantified 30 flavonoids in both MS and YDS rhizomes, among which 21 showed elevated abundances in the MS rhizome. Collectively, these results currently demonstrate a negative correlation between the expression of key lignin pathway genes and flavonoids accumulation across rhizome developmental stages.

From an energy allocation perspective, these findings indicate preferential partitioning of photoassimilates into structural biopolymers like lignin within YDS rhizome, thereby enhancing rhizome development. MS rhizome of *C. barometz*, being distal to the shoot apical meristem, enter a state of developmental quiescence, characterized by arrested lateral expansion and axial elongation. Such physiological constraints shift carbon allocation toward secondary metabolism, especially contributing to flavonoids accumulation. The biosynthesis of lignin and flavonoids in plants does not represent a simple antagonistic relationship (Li et al., 2010), but rather constitutes a dynamic adaptation balancing growth and defense (Yang et al., 2025; Zhang et al., 2021). Confirming a direct inverse relationship between flavonoid accumulation and lignin biosynthesis in MS rhizome necessitates additional molecular and phytochemical validation.

## Conclusion

Integrating broadly targeted metabolomics, transcriptome sequencing, and quantitative UPLC-MS/MS validation, we elucidated the secondary metabolite profile of the traditional Chinese medicine herb *Gou-ji*, its variation across growth years, and the underlying molecular mechanisms governing this metabolic reprogramming. Our analyses successfully identified 761 secondary metabolites, with phenolic acids and flavonoids collectively constituting over 50% of the detected profile. We demonstrated distinct stage-specific accumulation patterns: phenolic acids were predominant in juvenile (YDS) rhizome, alkaloids in intermediate (MDS) rhizome, and flavonoids in mature (MS) rhizome. Transcriptomic profiling revealed significant upregulation of flavonoid biosynthetic genes in MS rhizome, providing the molecular foundation for the observed metabolic shift. MS rhizome exhibited enhanced global transcriptional activity and significant upregulation of genes involved in the flavonoid biosynthesis pathway. Quantitative validation confirmed substantially higher levels of specific flavonoids—including (-)-epicatechin, apigenin-7-glucoside, and naringenin-7-glucoside—in MS compared to YDS rhizome. These findings indicated that prolonged growth duration is a critical factor contributing to flavonoid accumulation in the rhizome. Therefore, MS rhizome could be considered as a superior source of *Gou-ji* if flavonoids were established as the primary bioactive constituents underlying its traditional medicinal efficacy. Our results provide not only a phytochemical basis for understanding *Gou-ji*’s traditional efficacy but also valuable insights for quality assessment and determination of optimal harvest time for this medicinal herb.

However, the biological functions of the identified flavonoids and their key regulatory genes have not yet been experimentally validated in this study. Additionally, the lignin content in rhizomes of different ages was not determined, which would have provided more direct evidence for the competition between lignin and flavonoid biosynthesis. While omics studies provide a global perspective of the metabolome in *C. barometz*, establishing a causal link to its pharmacology remains a pressing challenge. Future research should focus on pharmacological validation of the bioactivity of the identified flavonoids and functional characterization of the key regulatory genes, which will provide crucial evidence for establishing flavonoids as quality markers and elucidate the molecular mechanisms underlying metabolic reprogramming during rhizome development.

## Data Availability

The datasets presented in this study can be found in online repositories. The names of the repository/repositories and accession number(s) can be found in the article/**Supplementary Material**.

## Supplementary material

Supplementary File 1 The prime pairs for qRT-PCR.

Supplementary File 2 The equations for standard curves calculating the concentration value of flavonoids.

Supplementary File 3 A list of all identified secondary metabolites in the rhizome of *C. barometz*.

Supplementary File 4 A list of secondary metabolites enriched in YDS rhizome in comparison with MDS and MS rhizome.

Supplementary File 5 A list of secondary metabolites enriched in MDS rhizome in comparison with YDS and MS rhizome.

Supplementary File 6 All metabolites included in the volcano plot for the pairwise comparison.

Supplementary File 7 All metabolites included in the volcano plot for the pairwise comparison.

Supplementary File 8 All enriched flavonoids included in the clustering heatmap for the pairwise comparison.

Supplementary File 9 All enriched metabolites included in the upset plot for the pairwise comparison.

Supplementary File 10 A list of 160 DEGs belonging to flavonoid-related GO terms.

Supplementary File 11 10 flavonoid-related GO terms and associated 160 DEGs.

Supplementary File 12 Flavonoid-related GO terms and associated DEGs across different comparison groups.

Supplementary File 13 Quantification of 185 flavonoids in YDS and MS rhizomes of *C. barometz*.

## References

[B1] Baranwal A. Aggarwal P. Rai A. Kumar N. (2022). Pharmacological actions and underlying mechanisms of catechin: a review. Mini Rev. Med. Chem. 22, 821–833. doi: 10.2174/1389557521666210902162120, PMID: 34477517

[B2] Besseau S. Hoffmann L. Geoffroy P. Lapierre C. Legrand M. (2007). Flavonoid accumulation in arabidopsis repressed in lignin synthesis affects auxin transport and plant growth. Plant Cell 19, 148–162. doi: 10.1105/tpc.106.044495, PMID: 17237352 PMC1820963

[B3] Chandra S. (1970). Vascular organization of the rhizome of Cibotium barometz. Am. Fern J. 60, 68–72. doi: 10.2307/1546933

[B4] Chen G. Y. Wang Y. F. Yu X. B. Liu X. Y. Chen J. Q. Luo J. . (2022). Network pharmacology-based strategy to investigate the mechanisms of Cibotium barometz in treating osteoarthritis. Evid Based Complement Alternat Med. 2022, 1826299. doi: 10.1155/2022/1826299, PMID: 35873632 PMC9303148

[B5] Chen H. Y. Yu Y. Sun Q. W. (2025). Integrated transcriptomic and metabolomic analyses uncover regulatory networks and metabolite dynamics in Cibotium barometz leaf development. Russ J. Plant Physiol. 72, 29. doi: 10.1134/S1021443724608498

[B6] Cui X. Wang Y. Yu G. He B. Huang L. Liu Y. . (2025). Integrated morphological observation, metabolomics, and transcriptomics to investigate the effect of growth years on the quality of Atractylodes macrocephala Koidz. BMC Plant Biol. 25, 912. doi: 10.1186/s12870-025-06958-0, PMID: 40660153 PMC12257821

[B7] Duan Y. Wu J. Wang F. Zhang K. Guo X. Tang T. . (2023). Transcriptomic and metabolomic analyses provide new insights into the appropriate harvest period in regenerated bulbs of Fritillaria hupehensis. Front. Plant Sci. 14. doi: 10.3389/fpls.2023.1132936, PMID: 36875619 PMC9975545

[B8] Felgines C. Texier O. Morand C. Manach C. Scalbert A. Régerat F. . (2000). Bioavailability of the flavanone naringenin and its glycosides in rats. Am. J. Physiol. Gastrointest Liver Physiol. 279, G1148–G1154. doi: 10.1152/ajpgi.2000.279.6.g1148, PMID: 11093936

[B9] Fraga C. G. Oteiza P. I. Galleano M. (2018). Plant bioactives and redox signaling: (-)-Epicatechin as a paradigm. Mol. Aspects Med. 61, 31–40. doi: 10.1016/j.mam.2018.01.007, PMID: 29421170

[B10] Freitas M. Ribeiro D. Janela J. S. Varela C. L. Costa S. C. Silva E. T. . (2024). Plant-derived and dietary phenolic cinnamic acid derivatives: Anti-inflammatory properties. Food Chem. 459, 140080. doi: 10.1016/j.foodchem.2024.140080, PMID: 38986205

[B11] Gambino G. Perrone I. Gribaudo I. (2010). A rapid and effective method for RNA extraction from different tissues of grapevine and other woody plants. Phytochem. Anal. 19, 520–525. doi: 10.1002/pca.1078, PMID: 18618437

[B12] Hazrati S. Mousavi Z. Nicola S. (2024). Harvest time optimization for medicinal and aromatic plant secondary metabolites. Plant Physiol. Biochem. 212, 108735. doi: 10.1016/j.plaphy.2024.108735, PMID: 38781639

[B13] Hu J. R. Zhang Huang L. L. Wu X. K. Spicer R. A. Quan C. . (2023). The first megafossil of Cibotium within its modern distribution. J. Palaeogeogr. 12, 96–106. doi: 10.1016/j.jop.2022.12.002

[B14] Huang D. Hou X. Zhang D. Zhang Q. Yan C. (2019). Two novel polysaccharides from rhizomes of Cibotium barometz promote bone formation via activating the BMP2/SMAD1 signaling pathway in MC3T3-E1 cells. Carbohydr Polym 231, 115732. doi: 10.1016/j.carbpol.2019.115732, PMID: 31888819

[B15] Huang D. Zhang M. Chen W. Zhang D. Wang X. Cao H. . (2018). Structural elucidation and osteogenic activities of two novel heteropolysaccharides obtained from water extraction residues of Cibotium barometz. Ind. Crops Prod 121, 216–225. doi: 10.1016/j.indcrop.2018.04.070

[B16] Jia X. H. Wang C. Q. Liu J. H. Li X. W. Wang X. Shang M. Y. . (2013). Comparative studies of saponins in 1-3-year-old main roots, fibrous roots, and rhizomes of Panax notoginseng, and identification of different parts and growth-year samples. J. Nat. Med. 67, 339–349. doi: 10.1007/s11418-012-0691-6, PMID: 22843418

[B17] Jiang R. H. Liang S. Q. Wu F. Tang L. M. Qin B. Chen Y. Y. . (2023). Phylogenomic analysis, cryptic species discovery, and DNA barcoding of the genus Cibotium in China based on plastome data. Front. Plant Sci. 14. doi: 10.3389/fpls.2023.1183653, PMID: 37346120 PMC10279961

[B18] Ju C. G. Xu G. Song Y. J. Zhao W. L. Jia T. Z. (2012). Comparison of the content of total phenolic acid in Cibotium barometz and its processed products from different areas. China J. Exp. Trad Med. Formulae 18, 24–26. doi: 10.13422/j.cnki.syfjx.2012.10.019

[B19] Kim Y. J. Joo S. C. Shi J. Hu C. Quan S. Hu J. . (2017). Metabolic dynamics and physiological adaptation of Panax ginseng during development. Plant Cell Rep. 37, 393–410. doi: 10.1007/s00299-017-2236-7, PMID: 29150823

[B20] Kim N. H. Lee J. Y. Kim C. Y. (2023). Protective role of ethanol extract of Cibotium barometz (Cibotium Rhizome) against dexamethasone-induced muscle atrophy in C2C12 myotubes. Int. J. Mol. Sci. 24, 14798. doi: 10.3390/ijms241914798, PMID: 37834245 PMC10573348

[B21] Kiokias S. Proestos C. Oreopoulou V. (2020). Phenolic acids of plant origin-a review on their antioxidant activity *in vitro* (O/W Emulsion Systems) along with their *in vivo* health biochemical properties. Foods 9, 534. doi: 10.3390/foods9040534, PMID: 32344540 PMC7231038

[B22] Li X. Bonawitz N. D. Weng J. K. Chapple C. (2010). The growth reduction associated with repressed lignin biosynthesis in Arabidopsis thaliana is independent of flavonoids. Plant Cell 22, 1620–1632. doi: 10.1105/tpc.110.074161, PMID: 20511296 PMC2899864

[B23] Li Y. Kong D. Fu Y. Sussman M. R. Wu H. (2020). The effect of developmental and environmental factors on secondary metabolites in medicinal plants. Plant Physiol. Biochem. 148, 80–89. doi: 10.1016/j.plaphy.2020.01.006, PMID: 31951944

[B24] Li L. Xie M. P. Sun H. Lu A. Q. Zhang B. Zhang D. . (2019). Bioactive phenolic acid-substituted glycoses and glycosides from rhizomes of Cibotium barometz. J. Asian Nat. Prod Res. 21, 947–953. doi: 10.1080/10286020.2018.1563076, PMID: 30693790

[B25] Lim T. K. (2016). “ Edible medicinal and non-medicinal plants,” in Modified Stems, Roots, Bulbs, vol. 10. ( Springer, Netherlands).

[B26] Liu W. Feng Y. Yu S. Fan Z. Li X. Li J. . (2021). The flavonoid biosynthesis network in plants. Int. J. Mol. Sci. 22, 12824. doi: 10.3390/ijms222312824, PMID: 34884627 PMC8657439

[B27] Liu H. Gao J. H. Xie Y. M. Li Y. Y. Li S. H. Zhang L. . (2020). Interpretation of expert consensus on Shujin Jianyao pills in clinical practice. China J. Chin. Materia Med. 45, 3336–3339. doi: 10.19540/j.cnki.cjcmm.20200229.502, PMID: 32726048

[B28] Livak K. J. Schmittgen T. D. (2001). Analysis of relative gene expression data using real-time quantitative PCR and the 2–ΔΔCT method. Methods 25, 402–408. doi: 10.1006/meth.2001.1262, PMID: 11846609

[B29] Min X. Zhu T. Hu X. Hou C. He J. Liu X. (2023). Transcriptome and metabolome analysis of isoquinoline alkaloid biosynthesis of Coptis chinensis in different years. Genes (Basel) 14, 2232. doi: 10.3390/genes14122232, PMID: 38137054 PMC10742649

[B30] Nett R. S. Dho Y. Tsai C. Passow D. Grundman J. M. Low Y. Y. . (2023). Plant carbonic anhydrase-like enzymes in neuroactive alkaloid biosynthesis. Nature 624, 182–191. doi: 10.1038/s41586-023-06716-y, PMID: 37938780 PMC10700139

[B31] Qin G. Pan D. Long Y. Lan H. Guan D. Song J. (2024). Chromosome-scale genome of the fern Cibotium barometz unveils a genetic resource of medicinal value. Horticulturae 10, 1191. doi: 10.3390/horticulturae10111191

[B32] Salehi B. Venditti A. Sharifi-Rad M. Kręgiel D. Sharifi-Rad J. Durazzo A. . (2019). The therapeutic potential of apigenin. Int. J. Mol. Sci. 20, 1305. doi: 10.3390/ijms20061305, PMID: 30875872 PMC6472148

[B33] Seyedi Z. Amiri M. S. Mohammadzadeh V. Hashemzadeh A. Haddad-Mashadrizeh A. Mashreghi M. . (2023). Icariin: A promising natural product in biomedicine and tissue engineering. J. Funct. Biomater 14, 44. doi: 10.3390/jfb14010044, PMID: 36662090 PMC9862744

[B34] Shen N. Wang T. Gan Q. Liu S. Wang L. Jin B. (2022). Plant flavonoids: Classification, distribution, biosynthesis, and antioxidant activity. Food Chem. 383, 132531. doi: 10.1016/j.foodchem.2022.132531, PMID: 35413752

[B35] Shi W. Wang Y. Li J. Zhang H. Ding L. (2007). Investigation of ginsenosides in different parts and ages of Panax ginseng. Food Chem. 102, 664–668. doi: 10.1016/j.foodchem.2006.05.053

[B36] Vogt T. (2010). Phenylpropanoid biosynthesis. Mol. Plant 3, 2–20. doi: 10.1093/mp/ssp106, PMID: 20035037

[B37] Wang W. Gao T. Yang H. Sun Y. Yang J. Zhou J. . (2024). The balance between lignin and flavonoid metabolism has a central role in the changes of quality in young shoots of the tea plant (Camellia sinensis). Sci. Hortic. 338, 113788. doi: 10.1016/j.scienta.2024.113788

[B38] Wang Q. Jiang Y. Mao X. Yu W. Lu J. Wang L. (2022). Integration of morphological, physiological, cytological, metabolome and transcriptome analyses reveal age inhibited accumulation of flavonoid biosynthesis in Ginkgo biloba leaves. Ind. Crops Prod 187, 115405. doi: 10.1016/j.indcrop.2022.115405

[B39] Wang T. Y. Li Q. Bi K. S. (2018). Bioactive flavonoids in medicinal plants: Structure, activity and biological fate. Asian J. Pharm. Sci. 13, 12–23. doi: 10.1016/j.ajps.2017.08.004, PMID: 32104374 PMC7032191

[B40] Wu Q. Yang X. W. (2009). The constituents of Cibotium barometz and their permeability in the human Caco-2 monolayer cell model. J. Ethnopharmacol 125, 417–422. doi: 10.1016/j.jep.2009.07.017, PMID: 19635547

[B41] Xie M. P. Li L. Sun H. Lu A. Q. Zhang B. Shi J. G. . (2017). Hepatoprotective hemiterpene glycosides from the rhizome of Cibotium barometz (L.). J. Sm. Phytochem. 138, 128–133. doi: 10.1016/j.phytochem.2017.02.023, PMID: 28262248

[B42] Xu X. R. Wang J. Y. Wang X. Y. Bi H. Z. Bai Q. X. Wang M. (2025). Botany, traditional uses, phytochemistry, pharmacology, processing, and applications of Cibotium barometz (L.) J. Sm.: A review. Fitoterapia. 186, 106843. doi: 10.1016/j.fitote.2025.106843, PMID: 40848860

[B43] Yan J. Zeng H. Chen W. Zheng S. Luo J. Jiang H. . (2023). Effects of tree age on flavonoids and antioxidant activity in Torreya grandis nuts via integrated metabolome and transcriptome analyses. Food Front. 4, 358–367. doi: 10.1002/fft2.211

[B44] Yan X. Zhang A. Guan Y. Jiao J. Ghanim M. Zhang Y. . (2024). Comparative metabolome and transcriptome analyses reveal differential enrichment of metabolites with age in panax notoginseng roots. Plants 13, 1441. doi: 10.3390/plants13111441, PMID: 38891250 PMC11175106

[B45] Yang J. Zhang Y. Jia J. Wang C. Fu Y. (2025). Flavonoid-lignin crosstalk: engineering metabolic flux for optimised plant growth and stress resilience. Plant Cell Environ.48(11), 8141–8160. doi: 10.1111/pce.70106, PMID: 40793933

[B46] Yin Q. Xiang L. Han X. Zhang Y. Lyn R. Yuan L. . (2025). The evolutionary advantage of artemisinin production by Artemisia annua. Trends Plant Sci. 30, 213–226. doi: 10.1016/j.tplants.2024.09.006, PMID: 39362811

[B47] Yu Y. G. Guo X. Y. Li X. Y. Dai D. D. Xu X. R. Ge X. J. . (2021). Organ- and age-specific differences of Dioscorea polystachya compounds measured by Uplc-Qtof/Ms. Chem. Biodivers 18, e2000856. doi: 10.1002/cbdv.202000856, PMID: 33295037

[B48] Yuan Y. Zuo J. Zhang H. Zu M. Liu S. (2022). Analysis of the different growth years accumulation of flavonoids in Dendrobium moniliforme (L.) Sw. by the integration of metabolomic and transcriptomic approaches. Front. Nutr. 9. doi: 10.3389/fnut.2022.928074, PMID: 36225877 PMC9549206

[B49] Zhang S. Yang J. Li H. Chiang V. L. Fu Y. (2021). Cooperative regulation of flavonoid and lignin biosynthesis in plants. Crit. Rev. Plant Sci. 40, 1–18. doi: 10.1080/07352689.2021.1898083

[B50] Zhang F. Zhang X. Luo Y. Li H. Qin X. (2022). Biosynthetic mechanisms of isoflavone accumulation affected by different growth patterns in Astragalus mongholicus products. BMC Plant Biol. 22, 410. doi: 10.1186/s12870-022-03769-5, PMID: 35996112 PMC9396891

[B51] Zhao X. Wu Z. X. Zhang Y. Yan Y. B. He Q. Cao P. C. . (2011). Anti-osteoporosis activity of Cibotium barometz extract on ovariectomy-induced bone loss in rats. J. Ethnopharmacol 137, 1083–1088. doi: 10.1016/j.jep.2011.07.017, PMID: 21782010

