# Supplementary material for: Integrated metabolomic and transcriptomic analyses reveal temporal dynamics of secondary metabolite accumulation in Cibotium barometz rhizome
Source: Front Plant Sci. 2025 Dec 5;16:1702726. doi: 10.3389/fpls.2025.1702726 (PMC12714926; doi:10.3389/fpls.2025.1702726)
Supplement: Supplementary file 14 [file Table14.docx]

**Legends for supplementary tables**

**Table S1.** The prime pairs for qRT-PCR

**Table S2.** The equations for standard curves calculating the concentration value of flavonoids.

**Table S3.** A list of all identified secondary metabolites in the rhizome of *C. barometz*.

**Table S4**. A list of secondary metabolites enriched in YDS rhizome in comparison with MDS and MS rhizome.

**Table S5.** A list of secondary metabolites enriched in MDS rhizome in comparison with YDS and MS rhizome.

**Table S6.** A list of secondary metabolites enriched in MS rhizome in comparison with YDS and MDS rhizome.

**Table S7.** All metabolites included in the volcano plot for the pairwise comparison.

**Table S8.** All enriched flavonoids included in the clustering heatmap for the pairwise comparison.

**Table S9.** All enriched metabolites included in the upset plot for the pairwise comparison.

**Table S10.** A list of 160 DEGs belonging to flavonoid-related GO terms.

**Table S11.** 10 flavonoid-related GO terms and associated 160 DEGs.

**Table S12.** Flavonoid-related GO terms and associated DEGs across different comparison groups.

**Table S13.** Quantification of 185 flavonoids in YDS and MS rhizomes of *C. barometz.*
