# Supplementary material for: Integrated metabolomic and transcriptomic analyses reveal temporal dynamics of secondary metabolite accumulation in Cibotium barometz rhizome
Source: Front Plant Sci. 2025 Dec 5;16:1702726. doi: 10.3389/fpls.2025.1702726 (PMC12714926; doi:10.3389/fpls.2025.1702726)
Supplement: Supplementary file 15 [file DataSheet1.docx]

**
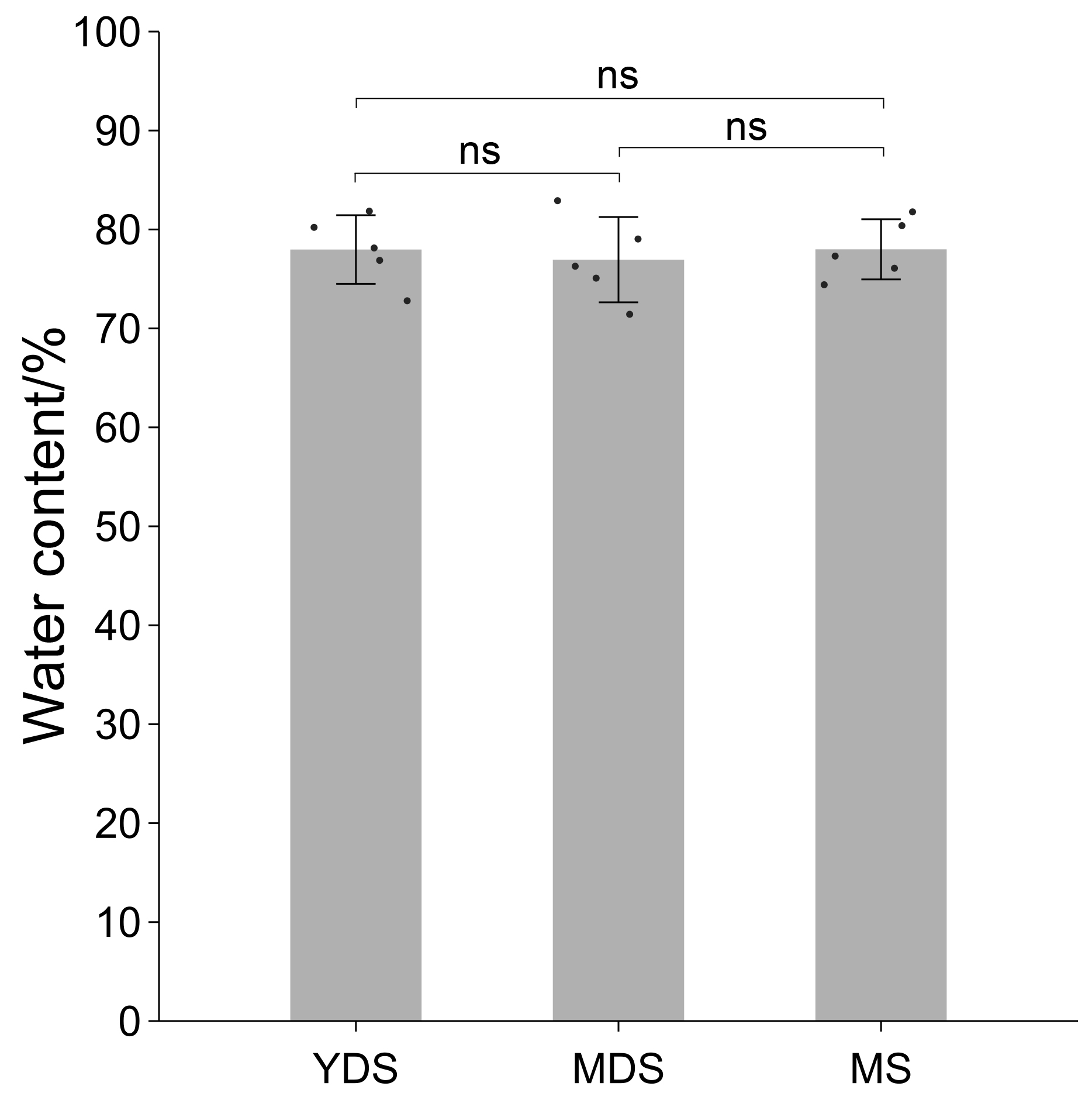
**

**Figure S1** The content of water in fresh rhizome of *C. barometz*. ns means no significance.


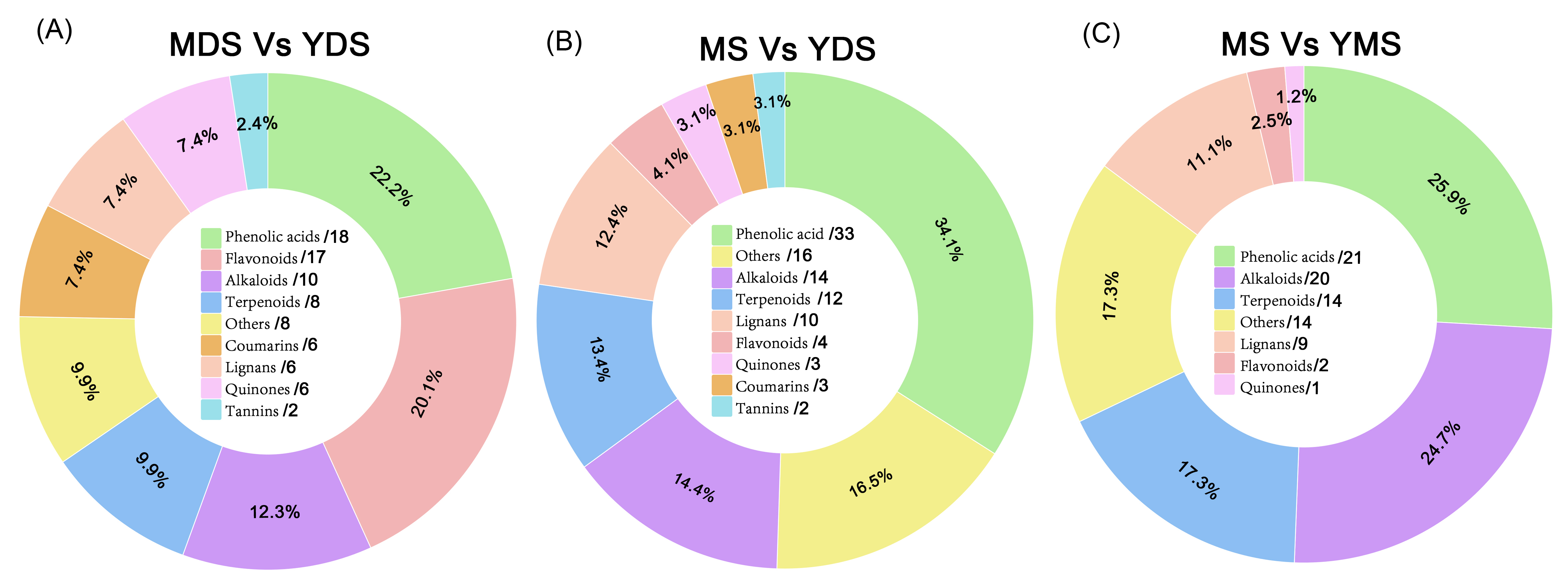


**Figure S2** Pie chart showing class-specific distribution of significantly reduced metabolites in pairwise comparison, each number behind forward-slash indicate the count of metabolites.


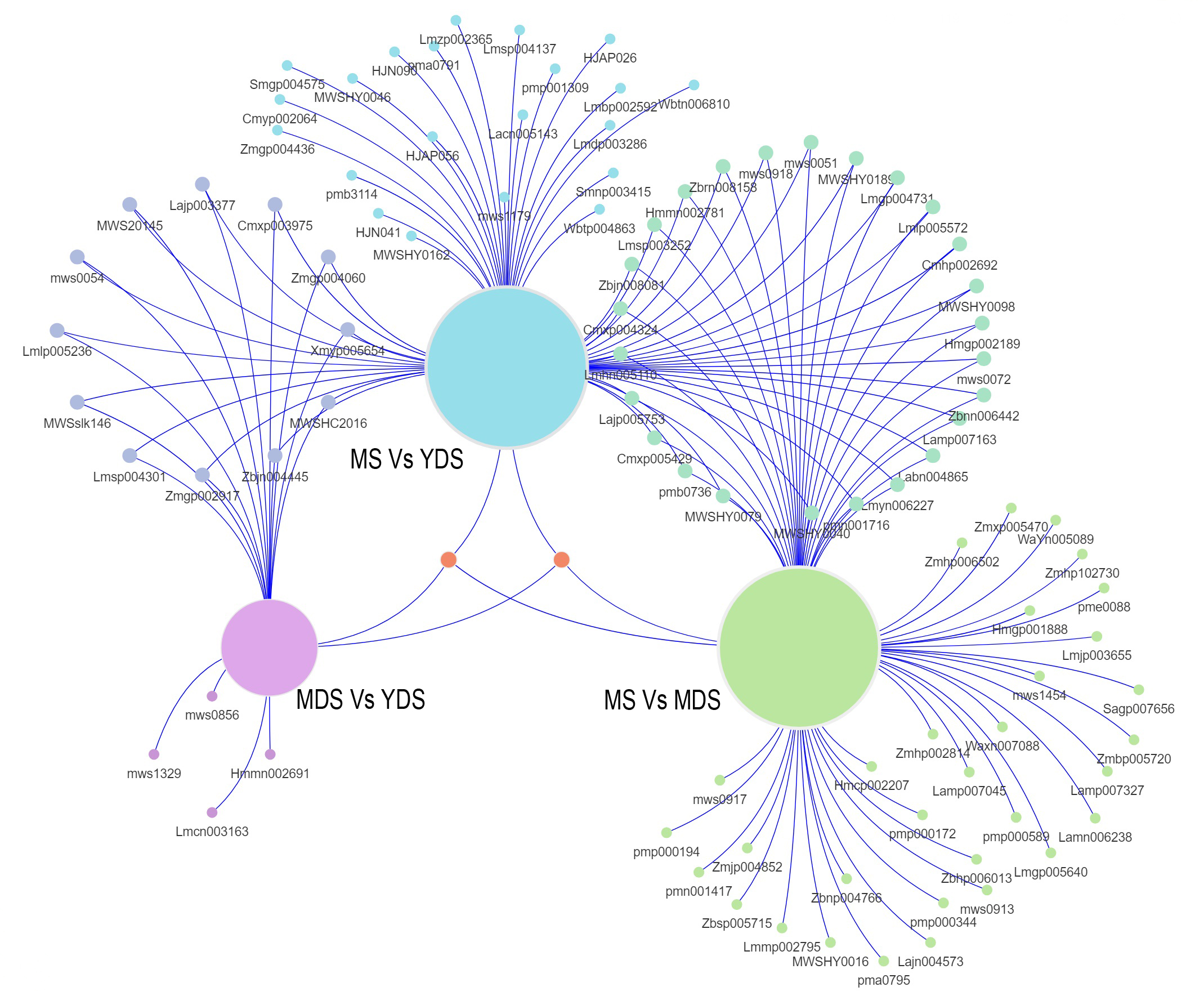


**Figure S3** The venn network for exhibiting the overlap of enriched flavonoids in the pairwise comparison.


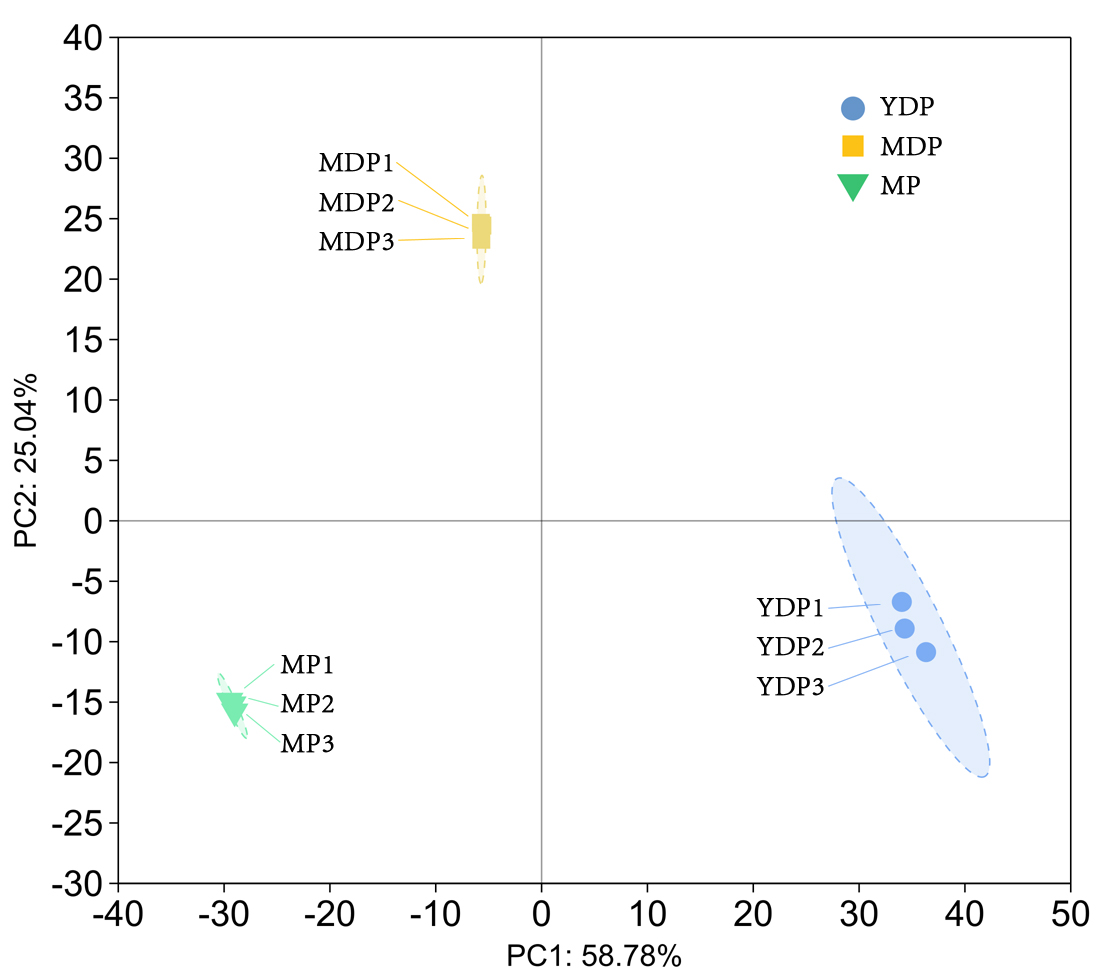


**Figure S4** Principal component analysis (PCA) of transcriptomes data. PC1 (58.78%) and PC2 (25.04%) capture 83.82% of total variance. Circle, square, and inverted triangle represents YDS, MDS, and MS rhizome, respectively. Samples clustered by developmental stage (n=3). Dashed ellipses: 95% confidence regions.

*
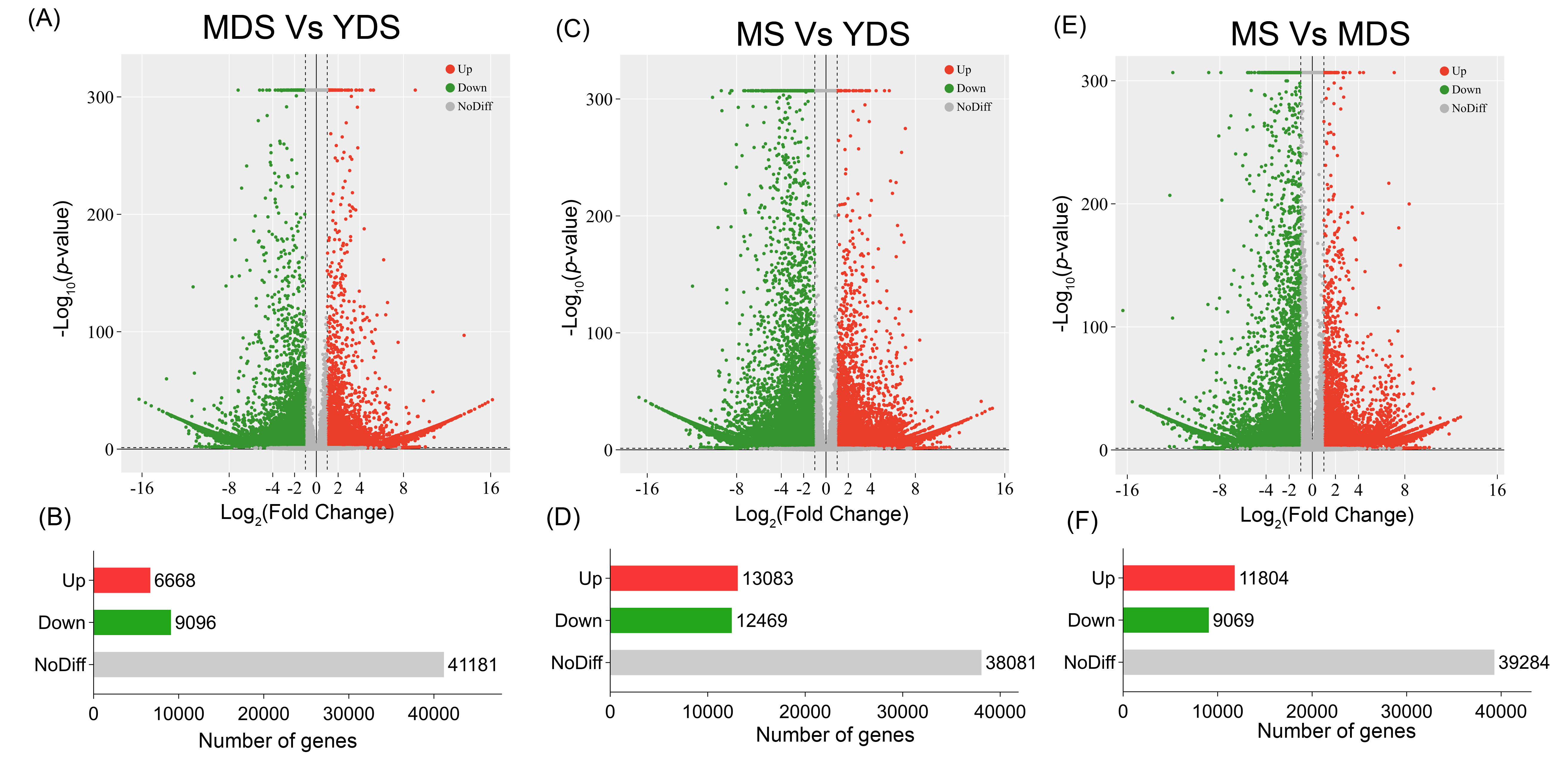
*

**Figure S5** (A, C, E) Volcano plot illustrating DEGs in distinct pairwise comparisons. Each point represents an annotated gene. The horizontal dashed line marks the significance threshold (-log₁₀(*p*-value) = 1.3, *p*-value < 0.05). Vertical dashed lines indicate a |log₂(FC)| > 1 threshold (FC > 2). Red and blue point denote significantly up-regulated and down-regulated gene, respectively; gray points represent genes with non-significant changes. (B, D, F) Histogram for the number of metabolites in pairwise comparisons.


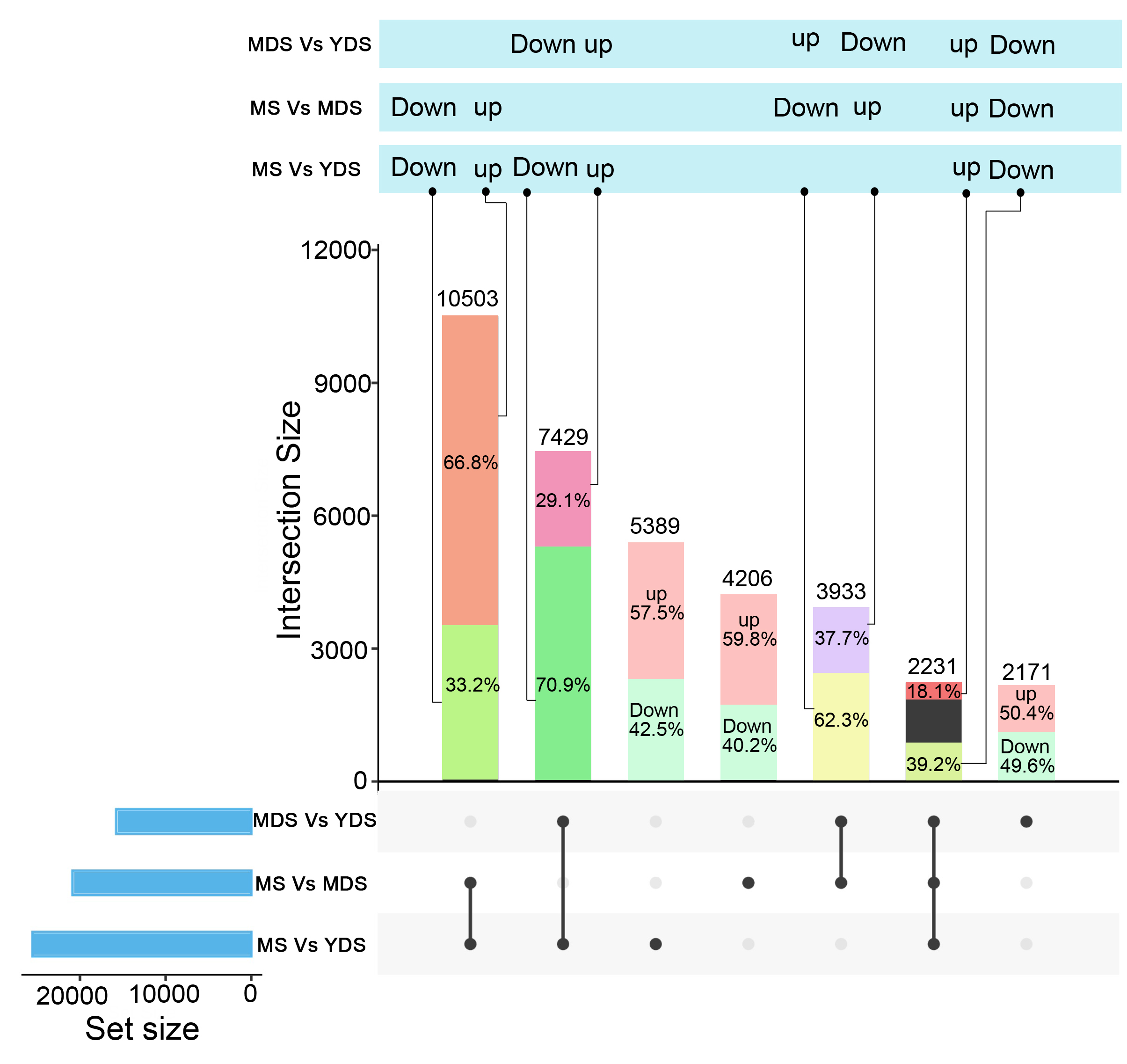


**Figure S6** An upset plot depicting the intersection of DEGs lists from three comparative analyses (MDS vs YDS, MS vs MDS, and MS vs YDS). The middle colorful bars indicate the size of the intersection for each specific combination of sets directly below it. The top matrix illustrates the percentage distribution of gene expression patterns across the intersection sets, as linked by the fine lines or signed on the bars. The bottom blue bars represent the size of individual set.


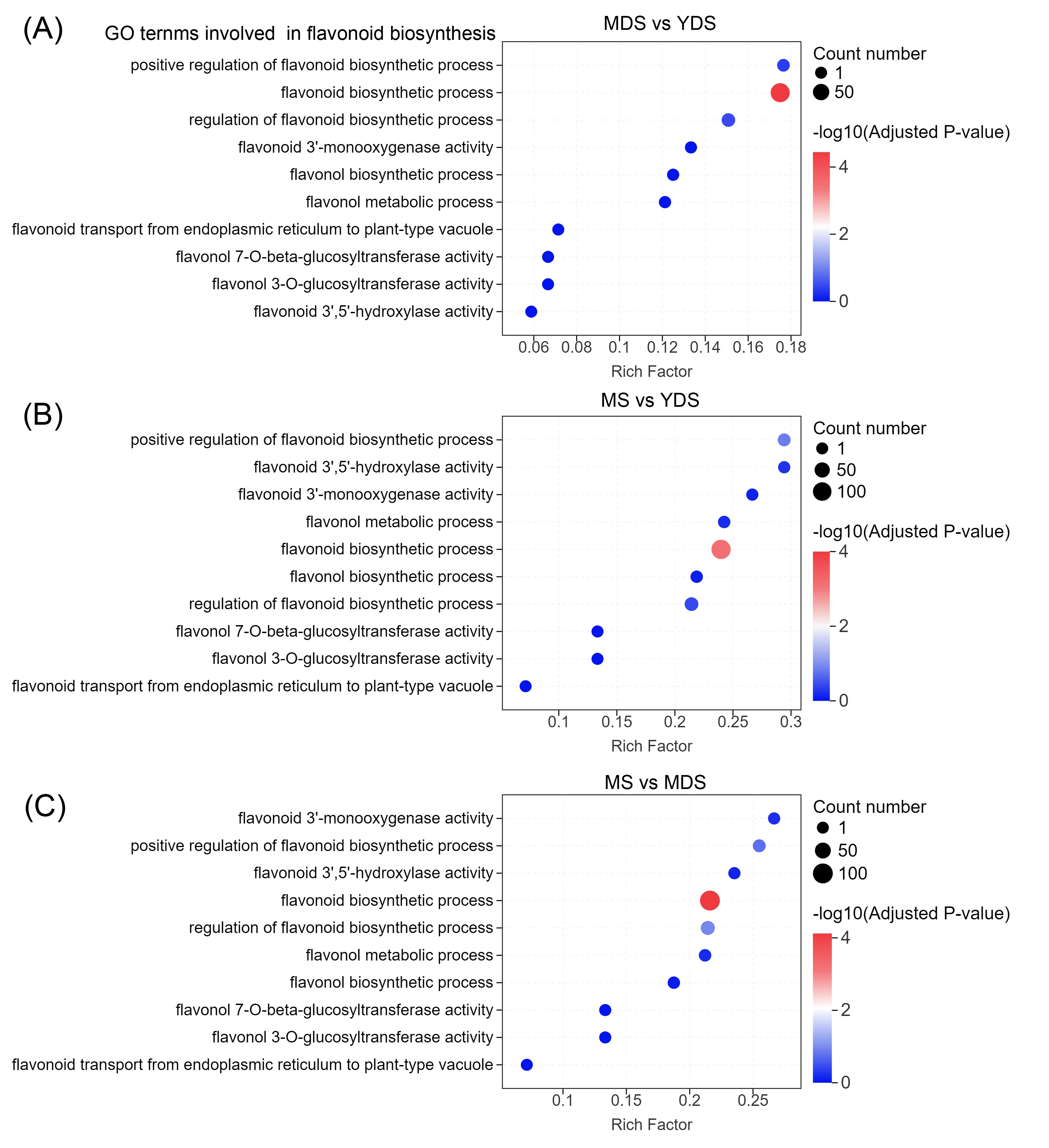


**Figure S7** The enrichment levels (Rich Factor) of flavonoid-related GO terms across different comparison groups.


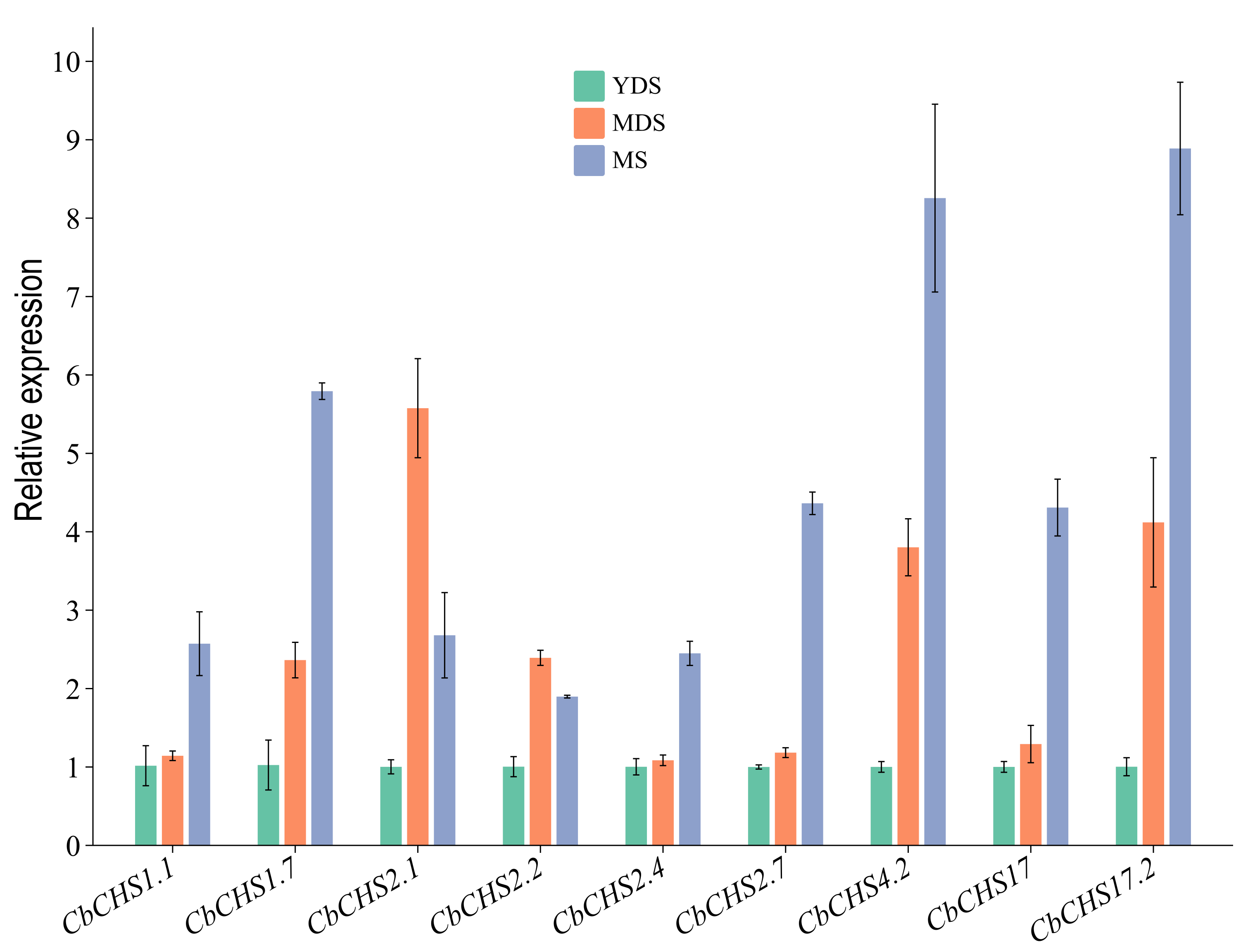


**Figure S8** The relative expression values of *CbCHS*s in the rhizome of *C. barometz*. *CbEF1A* was used as an internal control. Error bars represent mean + SD of three biological replicates. The following *CbCHS* genes were undetectable by qRT-PCR: *CbCHS1.6*, *CbCHS1.8*, *CbCHS2.6*, *CbCHS3.1*, *CbCHS17.3*.


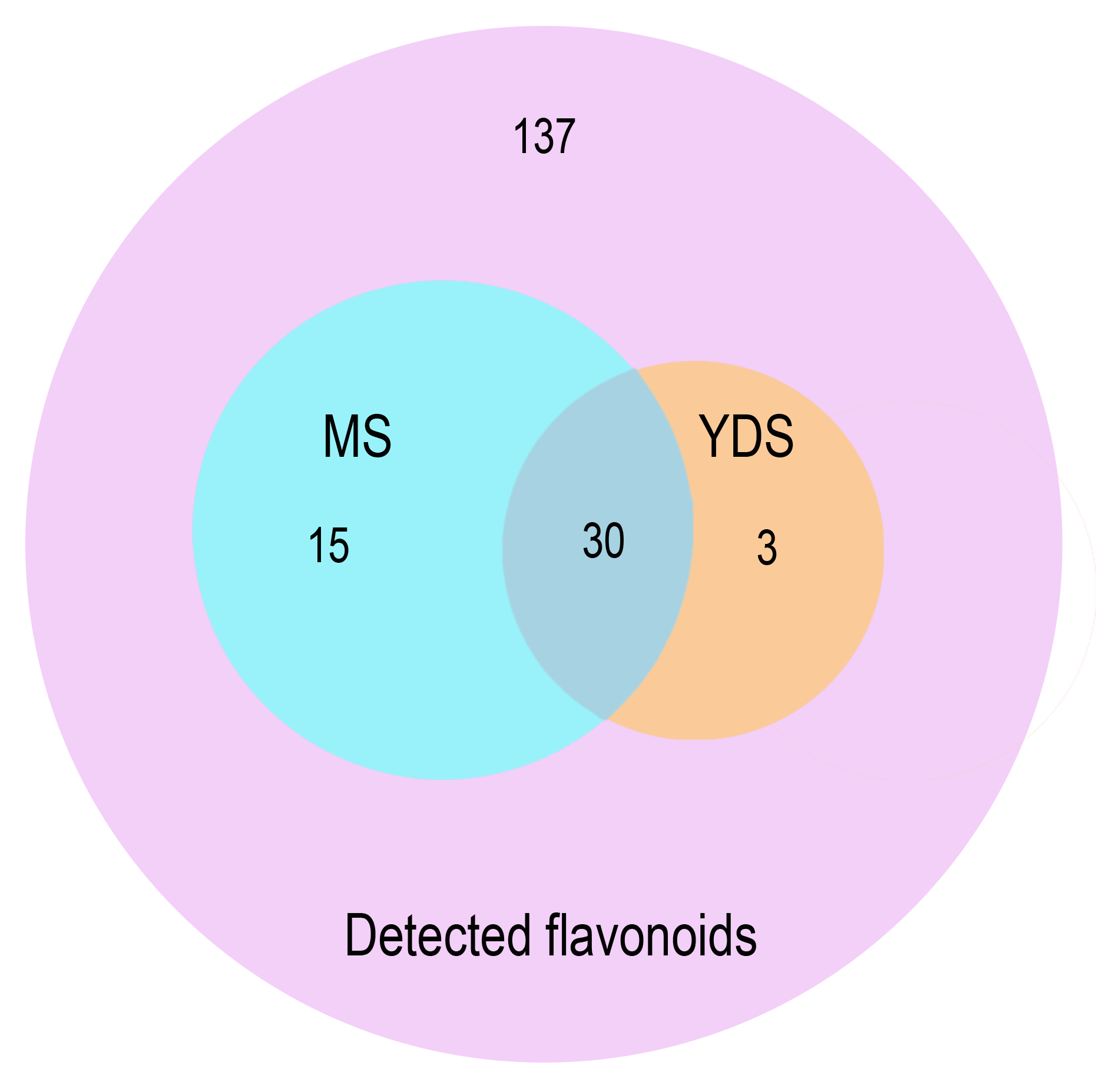


**Figure S9** The intersection venn diagram presenting the quantified flavonoids in YDS and MS rhizomes of *C. barometz.*
